# Effects of diacutaneous fibrolysis in patients with tension-type headache: A randomized controlled trial

**DOI:** 10.1371/journal.pone.0273877

**Published:** 2023-03-27

**Authors:** Sara Cabanillas-Barea, Silvia Pérez-Guillén, Carlos López-de-Celis, Jacobo Rodríguez-Sanz, Pablo Fanlo-Mazas, Andoni Carrasco-Uribarren

**Affiliations:** 1 Department of Physiotherapy, Faculty of Medicine and Health Sciences, Universitat Internacional de Catalunya, Sant Cugat del Vallès, Barcelona, Spain; 2 Department of Basic Sciences, Faculty of Medicine and Health Sciences, Universitat Internacional de Catalunya, Sant Cugat del Vallès, Barcelona, Spain; 3 Department of Physiatry and Nursery, Faculty of Health Sciences, University of Zaragoza, Zaragoza, Spain; IRCCS Medea: Istituto di Ricovero e Cura a Carattere Scientifico Eugenio Medea, ITALY

## Abstract

**Background:**

Manual therapy appears to be effective for the relief of tension-type headache (TTH), just as diacutaneous fibrolysis (DF) has shown to be a beneficial technique for the relief of symptoms in other dysfunctions. However, no studies have evaluated the potential beneficial effect of DF in TTH. The aim of this study is to analyze the effect of three sessions of DF in patients with TTH.

**Methods:**

Randomized controlled trial in 86 subjects (43 intervention/ 43 control group). The headache frequency, the headache intensity, the pressure pain thresholds (PPTs) at trapeziometacarpal joint, upper trapezius, suboccipital, frontal and temporal muscles, parietal sutures and the cervical mobility were measured at baseline, at the end of the third intervention and one-month after the last intervention.

**Results:**

Statistically significant differences with p values <0.05 were observed between groups in favor of the intervention group in the one-month follow-up in the following variables: headache frequency, headache intensity, flexion, extension, right and left side-bending, right and left rotation, PPTs in left trapeziometacarpal joint, right suboccipital muscle, right and left temporal muscle, left frontal muscle and right and left parietal.

**Conclusions:**

DF provides a beneficial effect in reducing headache frequency, relieving pain, and improving cervical mobility in patients with TTH.

## Introduction

Tension-type headache is the most common headache worldwide [1] and is well defined as a primary headache in the ICHD classification [2]. Symptoms can include bilateral pain in frontal and occipital regions, dull pain across the forehead, sides or back of the head and tenderness on the scalp or muscles of the neck, upper back, shoulders, and jaw [3, 4]. To establish a correct diagnosis, it is important is to discriminate between tension-type headache, medication-overuse-headache, and mild migraine without aura. The diagnosis of tension-type headache is based on the clinical characteristics of the pain and the associated symptoms, and is classified as infrequent, frequent, chronic, and probable. TTH is characterized by variable frequency and mild to moderate headache that is not associated with the typical debilitating migraine symptoms of nausea, vomiting, photophobia, and phonophobia [2].

The Eurolight project highlights that the total annual cost of headache, according to prevalence estimates, is €173 billion, divided between migraine (€111 billion; 64%), TTH (€21 billion; 12%), medication-overuse headache (37 billion euros; 21%) and other headaches (3 billion euros; 2%) [5].

The most common abnormal finding in these patients is the increased sensitivity of the pericranial structures; this sensitivity seems to increase uniformly throughout the cranial and cervical region, affecting skin, muscle, tendon, and fascial tissue [6]. In addition to the characteristics of pain, decreased cervical range of motion, decreased strength of the cervical muscles and excessive forward head position have been found [7].

Therapeutic approaches include pharmacological and non-pharmacological interventions [8]. For many individuals with tension-type headache, pharmacotherapy’s mainstays are simple analgesics and nonsteroidal inflammatory drugs [9]. Despite this, the European Federation of Neurological Societies guideline promotes non-pharmacologic therapies, having fewer side effects than pharmacological treatments [10]. Non-pharmacologic therapies may be a valid therapeutic option for subjects with headaches, despite their limited scientific basis [11], and the lack of evidence on the effectiveness of physical therapy options [12].

Physical therapy is the most used non-pharmacological treatment of tension-type headache and includes several treatment strategies such as manual therapy, postural exercise, relaxation, physical training, and mixed physiotherapy approaches [13, 14]. Manual therapies appear to be the most common non-medical treatment used to manage common recurrent headaches [4]. Within manual therapy techniques, soft tissue treatment resulted in a significant and beneficial effect on the pain intensity and frequency in these patients [14, 15].

Diacutaneous fibrolysis is a physiotherapeutic technique developed following Cyriax Deep Friction Massage principles [16]. A set of metallic hooks is used to achieve a more in-depth and more precise application than manually. A recent systematic review and meta-analysis published by Cadellans et al. [17] has shown that diacutaneous fibrolysis is an effective technique for improving symptoms and function in other musculoskeletal disorders such as shoulder pain [18, 19], chronic lateral epicondylalgia [20], patellofemoral pain [21] and carpal tunnel syndrome [22, 23].

The body of evidence supporting the clinical effectiveness of diacutaneous fibrolysis has increased in recent years. Nevertheless, to the best of the authors’ knowledge, no study to date has investigated the effects of diacutaneous fibrolysis in patients with tension-type headache. Therefore, the purpose of this randomized controlled trial was to evaluate the effects of diacutaneous fibrolysis on headache frequency, intensity, cervical pressure pain thresholds (PPTs) and cervical mobility in patients with tension-type headache. The hypothesis is that three sessions of diacutaneous fibrolysis would effectively reduce headache frequency and intensity, tissue sensitivity, and improve cervical range of motion in tension-type headache patients.

## Materials and methods

### Study design

The study design was a 2-group randomized controlled trial with pre-, post-intervention and one-month follow-up measurements. The allocation ratio was 1:1. The study was performed at University of Zaragoza from October 2015 to May 2018. A researcher not involved in the study randomized the intervention groups using a computer software (www.random.org). The results were placed in a concealed opaque envelope and were assigned to participants.

The study was conducted following the Declaration of Helsinki and the approval of the Clinical Research Ethics Committee of the Community of Aragon (CEICA), Spain (CEICA number: PI15/229) was obtained. All participants provided informed consent before their enrolment in the study. This clinical trial was carried out in the Faculty of Health Sciences facilities, University of Zaragoza, Spain; the center itself has a risk prevention model that could be activated if necessary. The participants remained in contact with their general practitioners and neurologists during the study to be able to report any incidents. (clinicaltrials.gov number: NCT03056131). This study followed the CONSORT criteria.

### Simple sizes calculation

The sample size was calculated based on the frequency of days with headache outcome, obtaining the highest number of subjects (43 subjects per group), making a total sample of at least 86 subjects. The sample size was calculated using the GRANMO 7.12 program with a α risk of 0.05, two-sided test and a β risk of 0.20. For frequency of headache, an estimated common standard deviation of 4.3 and a minimum expected difference in the follow-up of 2.7, and an estimated dropout rate of 0.07 were used [24].

### Subjects

Ninety-three subjects diagnosed with TTH by their neurologist or general practitioner in adherence to the International Classification of Headache Disorders (ICHD-3) criteria were recruited from primary care.

The inclusion criteria were age>18 and a TTH diagnosis. The ICHD-3 diagnostic criteria for chronic TTH were a headache occurring on ≥15 days/month on average for >3 months, and for the frequent episodic TTH diagnostic were headache occurring on at least 10 episodes of headache occurring on 1–14 days/month on average for >3 months. In both cases headache lasting from 30 minutes to 7 days, bilateral location, pressing/tightening quality, mild or moderate pain, not aggravated by routine physical activity, no nausea or vomiting and no more than one photophobia or phonophobia [2]. These diagnostic criteria continue to be used in the newly published guidelines for headache. Participants were excluded if they received physiotherapy treatment in the previous 3 months, if they had damaged skin, cutaneous lesions or vascular abnormalities in the cranio-cervical area, concomitant treatment with platelet antiaggregant agents, previous cervical or cranial surgery and patients with pending litigation or court claim.

### Procedure

**Fig 1:**
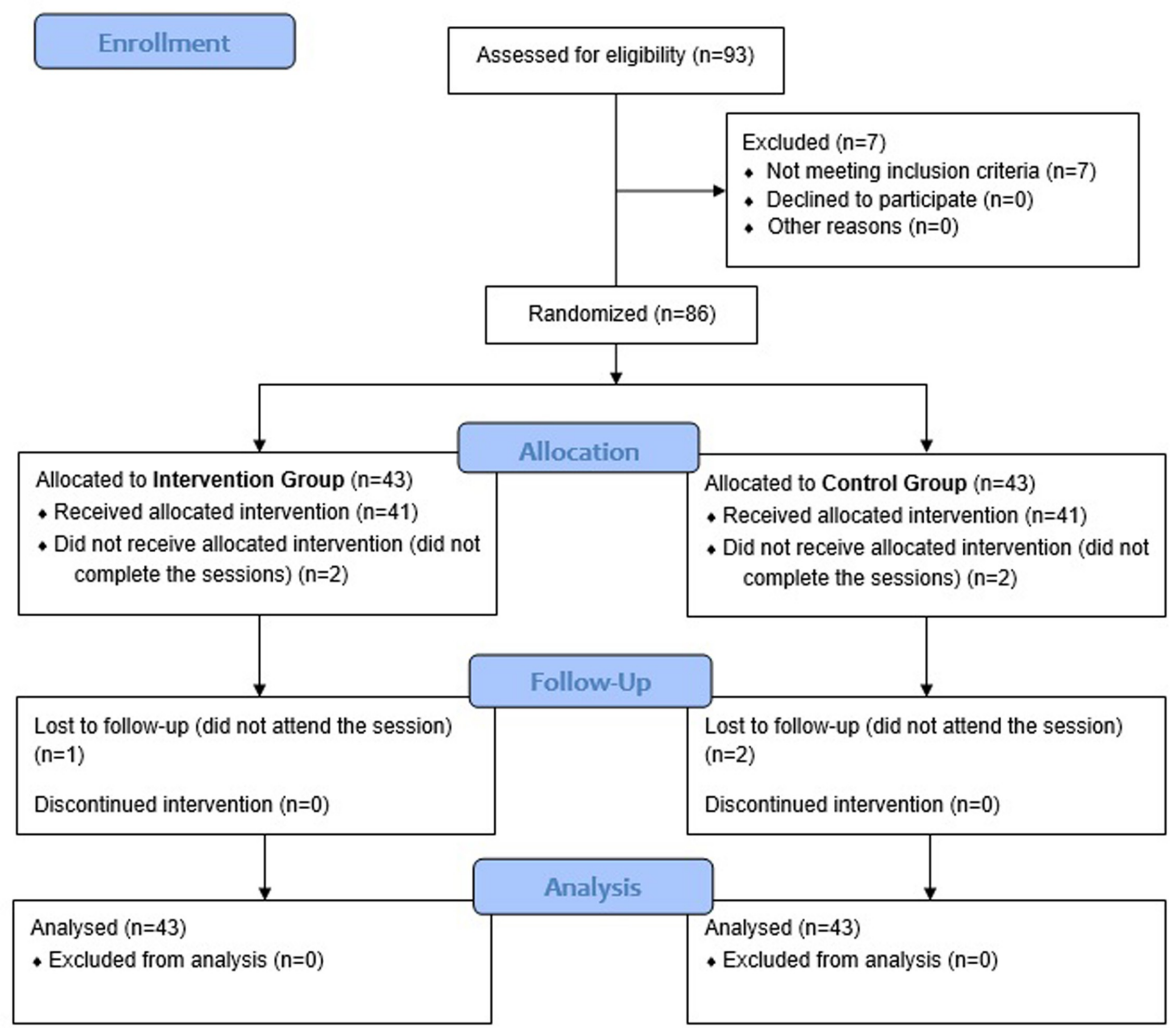
Study flow chart in accordance with the consolidated standards of reporting trials statement.

After baseline examination, participants were randomly allocated to the control (n = 43) or intervention (n = 43) group using a computer-generated sequence of numbers (simple randomization) using Microsoft Excel performed by an independent blinded investigator (Fig 1).

A physical therapist reviewed the selection criteria for each participant, provided them with the necessary information, and asked them to sign an informed consent form if they agreed. Another researcher made the assessments throughout the study at baseline (T0), post intervention (T1), and at 1 month follow-up (T2). This researcher was blinded to the assignment group of each subject during all the study. After the first evaluation, this last researcher gave each subject a sealed and opaque envelope in which the statistician had previously included the subject ‘s number and the assigned group. The physiotherapist who applied the treatment after opening the envelope was the only one who knew the group to which each participant belonged.

### Measurements

The primary outcome was the frequency of the episodes of headache per month. As secondary outcomes the headache intensity, the pressure pain thresholds and cervical mobility were measured.

For the frequency of headache episodes a simple headache diary was used to record the days with TTH per 4 weeks where the patient should indicate for each day whether or not a headache was present. This variable was recorded at times T0 and T2.

The headache intensity was registered with a visual analogue scale, which is a valid and reliable tool for measuring headache pain intensity [25]. A 10 cm vertical line was used, with a descriptor at each end of the line ("no pain" and "worst pain imaginable"). The headache intensity was recorded in four different instants: actual pain, usual pain, worse and better in the last month.

Pressure pain threshold was measured using a handheld pressure algometer (Somedic AB, Farsta, Sweden) at different cervical region sites to reflect localized hyperalgesia and on the hand to reflect distal hyperalgesia. The probe (1 cm2) was placed right angle to the skin. The pressure was applied at a rate of 30 KPa/s, and participants were instructed to indicate when the sensation changed from a sensation of pressure to the first sensation of pain. The subject was in a supine position, and the pressure pain thresholds were assessed in trapeziometacarpal joint, suboccipital muscles, frontal muscles, temporal muscles, and parietal suture. All sites were first located and marked, topographical pressure pain points were mapped following previous studies description [26, 27]. The test sites on the neck and head were located based on bony landmarks. Patients press the button of the digital algometer at the precise moment that pressure sensation changed to pain. The reliability of this measurement is high (ICC = 0.91–0.97) [28].

The CROM (floating compass; Plastimo Airguide, Inc, Buffalo Groove, IL) device was used to measure the range of movement of the cervical spine, which has shown to have good intra- and interexaminer reliability (ICC > 0.80) in all the measurements [29]. The general active range of motion of the cervical spine was assessed in a sitting position with the back resting on the chair’s backrest. Flexion, extension, left and right side-bending and left and right rotation were registered. The flexion and extension of the upper cervical spine (UCS) were measured to stand with the head resting on a wall [30].

The Global Rating of Change Scale (GROC-Scale) was rated according to the question: “Compared to prior inclusion in the study, how much has your headache problem changed?” (Seven for improvement, seven for deterioration and one for without changes). The minimal clinically significant difference in patients with headache is ±4 on the GROC-scale. After the registration, the results were grouped into three categories: without clinical change, with clinical worsening and with clinical improvement [31].

All these variables were recorded in T0, T1 and T2, except for the frequency, the visual analogue scale “worst moment”, “best moment” and “usual pain” that were registered in T0 and T2. The GROC-scale was assessed at T1 and T2 follow-ups.

### Intervention

The intervention had a 1-week duration, with three face-to-face sessions of diacutaneous fibrolysis. The intervention was about 30-minute-long and was applied with at least 1-day separation between sessions. The physiotherapist who applied the intervention had more than ten years of experience in using the technique.

**Fig 2:**
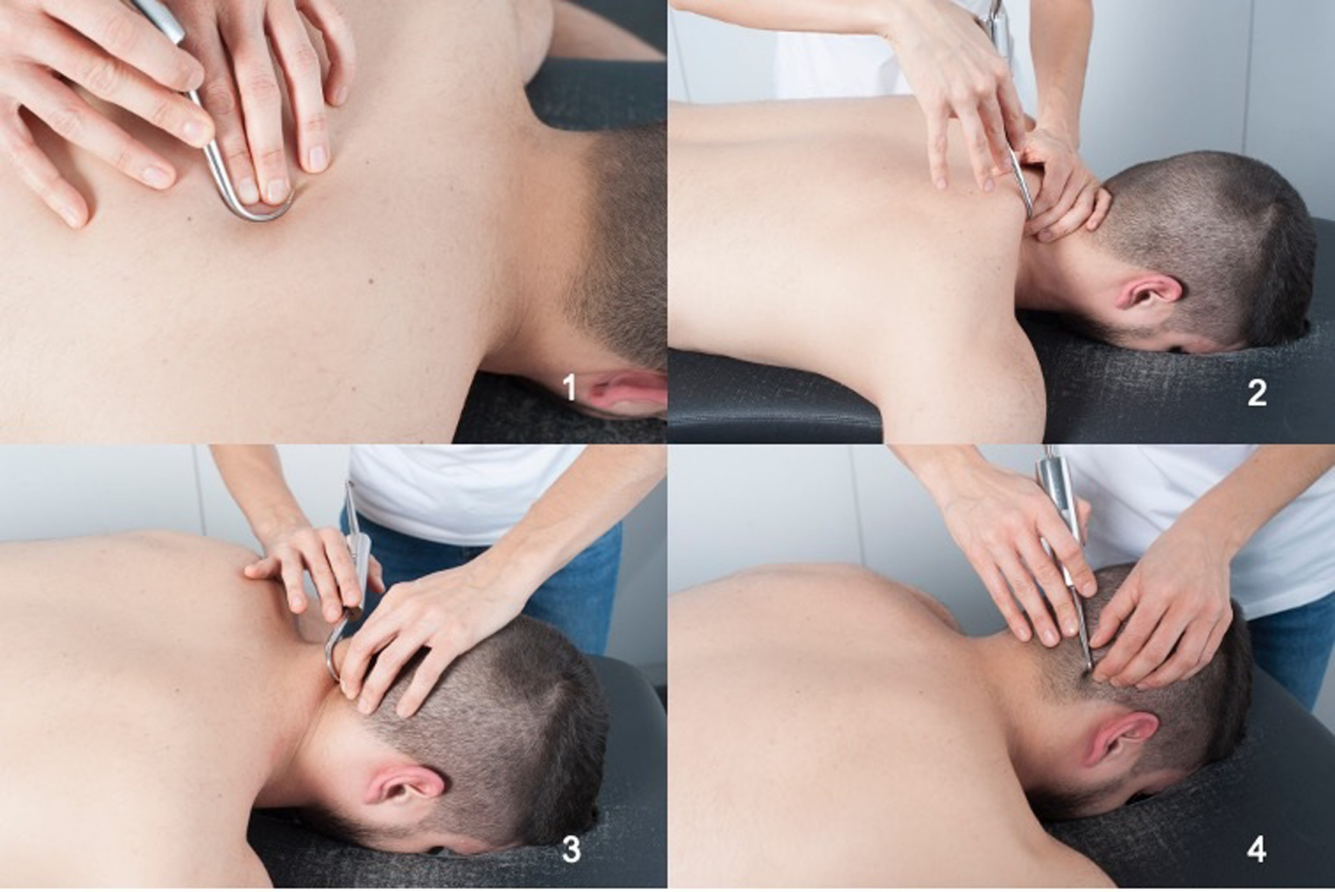
Diacutaneous fibrolysis techniques. 1. Treating painful points by pressure inhibition, 2 and 3. Separating different muscle planes and 4. Scratching tendon attachments.

The diacutaneous fibrolysis was performed in the intermuscular septum between muscles with an anatomical or functional relationship with the cervical spine. Those muscles were trapezius, levator scapulae, splenius cervicis, splenius capitis, and sternocleidomastoid. The technique was also applied on the bony edges of the dorsal spinous processes, the scapula, and the base of the skull. The diacutaneous fibrolysis intervention was applied in a centripetal direction towards the head (pain localization), starting at a distance from the thoracic region, continuing through the scapular region, and finally ending in the cervical and cranial region. The diacutaneous fibrolysis treatment involved three main techniques: treating painful points by pressure inhibition, separating different muscle planes, and scratching tendon attachments (Fig 2). The scratching technique was performed with the hook on the surface of the thoracic and cervical spinous processes, the scapular spine, the acromion, and the occipital line. Mobilization of the edge of the lower trapezius and the upper trapezius was performed with the hook. As well as the edge of the splenius muscles with the levator scapulae and the sternocleidomastoid muscle. Pressure inhibition was performed on the trapezius on latissimus dorsi and trapezius on rhomboid. The patient was changing position (supine, lateral and prone position) depending on the area to be treated.

The control group remained in the supine position for 15 minutes without receiving intervention. The placebo effect has already been studied with the simulated diacutaneous fibrolysis technique [18]. A sham technique was avoided as in other studies that have applied sham diacutaneous fibrolysis, when participants experienced a worsening of their symptoms [22].

Participants in the control group were offered to receive the treatment techniques upon completion of the study.

### Statistical analysis

The Statistical analysis was conducted with the SPSS 20.0 package (IBM, Armonk, New York) according to an intention to treat approach. The mean and standard deviation were calculated for each variable. To examine at baseline between group differences patient’s characteristic one-factor ANOVA or Mann Whitney-U test following the normally or non-normally distributed data and Chi-Square test was used. To determine the normal distribution of quantitative data the Kolmogorov-Smirnov test was used.

Linear mixed-model with repeated-measures were analyzed, the intervention effect over time were modeled to primary and secondary outcome measures, the significant level was set at (p < 0.05) The normal distribution of residuals was analyzed prior to carrying out the analysis. The random effects were modeled on individuals, and the fixed model on groups (Control group and Intervention group), time (Baseline, postintervention, and one-month follow-up) and group x time. All participants were included at the analysis because the mixed-model estimates values for missing data [32]. Bonferroni adjustments were used when the group x time or group were significant and change scores for post-intervention and one-month follow up comparing with baseline.

## Results

Ninety-three subjects were recruited between October 2015 to May 2018. Eighty-six met the selection criteria. Seventy-nine participants completed the study (57 females and 22 males, 38.35 age ±15.78), and seven dropped out (four did not complete the intervention protocol, and three did not attend the evaluation session). Subjects were randomly allocated to one group or another and were comparable at the baseline (*p* > 0.05) (Table 1).

**Table 1:** Subject demographic characteristics at baseline.

	Control group	Intervention group
Gender (Male/Female)	12/31	12/31
Age (years)	39.49 ± 16.26	37.25 ± 15.41
Weight (kg)	67.28 ± 13.33	67.27 ± 13.95
Height (m)	1.68 ± 0.06	1.67 ± 0.08
Age of headache onset (years)	26.13 ± 15.75	25.85 ± 16.05
Headache frequency (days)	13.26 ± 12.39	13.28 ± 11.97
Actual headache (VAS)	2.04 ± 1.76	1.75 ± 1.74
Usual headache (VAS)	2.99 ± 1.46	3.42 ± 0.90
Worse headache (VAS)	6.14 ± 1.71	6.27 ± 1.64
Better headache (VAS)	0.54 ± 1.05	0.55 ± 0.91
Pharmacological treatment		
NSAIDs	16 (41%)	18 (45%)
Acetaminohen and NSAIDs	7 (18%)	8 (20%)
Acetaminophen	16 (41%)	14 (35%)

Abbreviations: VAS: Visual analogue scale

The linear mixed-model showed a significant time-by-group results for frequency of headache (F = 29.10; *p* < 0.001). At baseline the headache frequency in control group was 13.26± 12.39 day/2weeks, and in the intervention group of 13.28 ± 11.97 day/2weeks. The intervention group revealed a greater decrease than the control group (Δ -9.66; -5.28 to -14.04) at T2 follow-up (Table 2).

**Table 2:** Outcome data headache frequency and intensity.

*Outcome*	Control group	Intervention group	*Between group-change score*
	(*n* = 43)	(*n* = 43)	
*Frequency of Headache*			
Baseline	13.26 ± 12.39	13.28 ± 11.97	
One-month follow-up T2	15.49 ± 11.98	5.82 ± 6.99	
Within-group changed T2	2.31 (0.11, 4.38)	- 7.45 (-10.38, -4.52)	9.66 (5.28, 14.04)
*Actual Headache VAS*			
Baseline	2.04 ± 1.77	1.75 ± 1.74	
Post-intervention T1	2.52 ± 2.62	0.74 ± 1.22	
Within-group changed T1	0.47 (-1.35, 0.39)	-1.01 (-0.32, -1.69)	1.77 (0.86, 2.68)
One-month follow-up T2	1.82 ± 2.08	0.86 ± 1.55	
Within-group changed T2	0.22 (-0.60, 1.44)	-0.88 (-0.18, -1.58)	0.95 (0.13, 1.77)
*Usual Headache VAS*			
Baseline	2.99 ± 1.46	3.42 ± 1.81	
One-month follow-up T2	3.13 ± 1.87	1.66 ± 1.43	
Within-group changed T2	0.13 (0.30, 0.57)	-1.76 (-1.17, -2.36)	1.47 (0.41, 2.48)
*Worse Headache VAS*			
Baseline	6.14 ± 1.72	6.27 ± 1.64	
One-month follow-up T2	5.91 ± 2.11	4.46 ± 2.50	
Within-group changed T2	-0.23 (-0.99, 0.52)	-1.81 (-2.63, 0.98)	1.77 (0.86, 2.69)
*Better Headache VAS*			
Baseline	0.54 ± 1.05	0.56 ± 0.91	
One-month follow-up T2	0.48 ± 0.90	0.18 ± 0.48	
Within-group changed T2	- 0.46 (-0.24, -0.15)	-0.37 (-0.65, -0.10)	0.31 (-0.01, 0.64)

A significant decrease in favour of the intervention group was observed in the linear mixed-model analysis at time-by-group analysis in the worse intensity (F = 8.20; *p* < 0.005) and in the usual intensity (F = 26.90; *p* < 0.001) of headache. In the intervention group the worse intensity of headache decrease more than the control group (Δ -1.45; -0.41 to -2.49) at T2 follow up. The usual intensity of headache showed a greater decrease in the intervention group (Δ -1.47; -0.73 to 2.22) (Table 2).

The mobility of cervical spine showed an improvement in favour the intervention group in all movements, the linear mixed-model showed a significant time-by-group results for flexion (F = 13.10; *p* < 0.001), extension (F = 30.34; *p* < 0.001), right side-bending (F = 26.56; *p* < 0.001), left side-bending (F = 20.37; *p* < 0.001), right rotation (F = 14.25; *p* > 0.001), left rotation (F = 19.47; *p* < 0.001), UCS flexion (F = 15.57; *p* < 0.001), UCS extension (F = 9.66; *p* > 0.003). The results between group and within group for T1 and T2 are shown in the Table 3.

**Table 3:** Outcome data range of movement.

*Outcome*	Control group	Intervention group	*Between group-change score*
	(*n* = 43)	(*n* = 43)	
*Flexion*			
Baseline	51.36 ± 10.43	47.55 ± 14.12	
Post-intervention T1	45.64 ± 10.13	53. 27 ± 11.63	
Within-group changed T1	-5.72 (-9.36, -2.07)	5.72 (0.82, 10.63)	-7.63 (-12.52, -2.74)
One-month follow-up T2	46.77 ± 11.23	52.27 ± 12.00	
Within-group changed T2	-4.59 (-4.25, 1.99)	4.72 (0.14, 9.31)	-5.51 (-10.72, 0.29)
*Extension*			
Baseline	55.54 ± 12.61	50.60 ± 11.46	
Post-intervention T1	50.46 ± 11.64	59.32 ± 14.24	
Within-group changed T1	-5.08 (-9.60, -0.56)	8.72 (4.13, 13.32)	-8.86 (-14.70, -3.02)
One-month follow-up T2	49.54 ± 11.45	57.00 ± 12.74	
Within-group changed T2	-6.00 (-9.90, -2.10)	6.40 (2.34, 10.64)	-7.46 (-12.89¡8, -2.04)
*Right rotation*			
Baseline	57.54 ± 9.21	56.55 ± 9.23	
Post-intervention T1	56.54 ± 9.07	63.30 ± 7.76	
Within-group changed T1	-1.00 (-4.32, 2.32)	6.75 (3.32, 10.18)	-6.68 (-10.54, -2.98)
One-month follow-up T2	55.25 ± 9.78	61.30 ± 7.44	
Within-group changed T2	-2.28 (-5.66, 1.10)	4.75 (1.53, 7.97)	-6.04 (-9.93, -2.16)
*Left rotation*			
Baseline	59.69 ± 9.33	58.72 ± 8.48	
Post-intervention T1	57.05 ± 8.40	65.62 ± 9.21	
Within-group changed T1	-2.64 (-5.97, 0.67)	6.90 (3.21, 10.59)	-8.57 (-12.53, -4.62)
One-month follow-up T2	56.69 ± 9.25	64.15 ± 8.86	
Within-group changed T2	-3.00 (-6.13, 0.13)	5.42 (1.83, 9.02)	-7.46 (-11.51, -3.40)
*Right side bending*			
Baseline	32.69 ± 8.90	33.02 ± 8.35	
Post-intervention T1	31.51 ± 7.50	38.60 ± 8.47	
Within-group changed T1	-1.18 (-3.16, 0.80)	5.58 (2.79, 8.36)	-7.08 (-10.67, -3.50)
One-month follow-up T2	29.54 ± 8.65	36.62 ± 9.53	
Within-group changed T2	-3.15 (-5.37, -0.94)	3.60 (1.19, 6.01)	-7.09 (-11.16, -3.01)
*Left side bending*			
Baseline	34.77 ± 8.13	33.95 ± 7.86	
Post-intervention T1	33.95 ± 7.06	40.62 ± 8.83	
Within-group changed T1	-0.82 (-3.06, 1.42)	6.67 (4.46, 8.89)	-6.68 (-10.26, -3.09)
One-month follow-up T2	34.00 ± 7.83	38.75 ± 8.29	
Within-group changed T2	-0.77 (-3.23, 1.70)	4.80 (2.92, 6.67)	-4.75 (-8.35, -1.15)
*Flexion UCS*			
Baseline	-0.85 ± 10.17	-2.20 ± 8.89	
Post-intervention T1	-2.56 ± 8.89	3.72 ± 9.51	
Within-group changed T1	-1.72 (-3.75, 0.32)	5.92 (3.70, 8.15)	-6.29 (-10.88, -1.70)
One-month follow-up T2	-2.41 ± 10.13	1.10 ± 9.80	
Within-group changed T2	-1.56 (-3.52, 0.40)	3.30 (0.93, 5.67)	-3.51 (-7.97, 0.95)
*Extension UCS*			
Baseline	35.92 ± 10.00	34.92 ± 6.80	
Post-intervention T1	35.13 ± 8.25	38.60 ±6.20	
Within-group changed T1	-0.79 (-3.38, 1.79)	3.67 (1.65, 5.70)	-3.47 (-6.73, -0.21)
One-month follow-up T2	35.90 ± 8.96	38.80 ± 6.31	
Within-group changed T2	-0.02 (-2.27, 2.22)	3.87 (1.68, 6.07)	-2.90 (-6.37, 0.56)

The linear mixed-model showed a significant time-by-group results for PPT in left trapeziometacarpal joint (F = 13.53; *p* < 0.001), right suboccipital muscle (F = 7.34; *p* < 0.008), right (F = 5.50; *p* < 0.022) and left (F = 8.43; *p* < 0.005) temporal muscle, left frontal muscle (F = 7.34; *p* < 0.008), and right (F = 6.66; *p* < 0.012) and left (F = 3.96; *p* < 0.05) parietal suture. At Table 4 are shown the data of the T1 and T2 follow-ups.

**Table 4:** Outcome data pressure pain threshold.

*Outcome*	Control group	Intervention group	*Between group-change score*
	(*n* = 14)	(*n* = 14)	
*Right Trapeziometacarpal joint*			
Baseline	385.62 ± 145.53	372.78 ± 138.83	
Post-intervention T1	332.64 ± 127.08	351.30 ± 166.62	
Within-group changed T1	-52.97 (-109.57, -3.62)	-21.47 (-63.26, 20.31)	-18.66 (-85.17, 47.85)
One-month follow-up T2	372.74 ± 1301.4	419.13 ± 192.55	
Within-group changed T2	- 12.87 (-79.03)	46.35 (-0.29, 92.99)	-46.38 (-120.20, 27.44)
*Left Trapeziometacarpal joint*			
Baseline	427.05 ± 144.57	380.68 ± 136.64	
Post-intervention T1	343.67 ± 123.27	355. 27 ±153.61	
Within-group changed T1	-83.38 (-130.51, -36.26)	-25.40 (-23.01, 73.81)	-11.61 (-74.10, 50.89)
One-month follow-up T2	365.92 ± 115.70	417.23 ± 187.24	
Within-group changed T2	-61.13 (-102.44, -19.82)	36.55 (-15.28)	-51.30 (-121.24, 18.64)
*Right Suboccipital muscles*			
Baseline	207.31 ± 80.19	206.38 ± 111.27	
Post-intervention T1	179.31 ± 91.53	210.98 ± 104.91	
Within-group changed T1	-28.00 (-49.61, -6.40)	4.60 (-28.13, 37.33)	-31.67 (-75.82, 12.49)
One-month follow-up T2	188.46 ± 81.37	235.90 ± 113.40	
Within-group changed T2	-18.85 (-43.45, 5.76)	29.52 (-7.05, 66.55)	-47.44 (-91.76, -3.12)
*Left Suboccipital muscles*			
Baseline	213.00 ± 81.34	212.01 ± 103.52	
Post-intervention T1	176.28 ± 64.96	206.55 ± 92.32	
Within-group changed T1	36.72 (-67.07, -6.37)	-5.45 (-23.61, 13.76)	-30.27 (-66.11, 5,58)
One-month follow-up T2	193.31 ± 81.32	220.58 ± 107.60	
Within-group changed T2	-19.69 (-53.15, 13.76)	8.57 (-22.07, 39.22)	-27.27 (-70.08, 15.54)
*Right Frontal muscles*			
Baseline	267.13 ± 113.10	280.05 ± 129.24	
Post-intervention T1	264.59 ± 115.42	288.20 ± 140.99	
Within-group changed T1	-2.54 (-41.69, 36.61)	8.15 (-27.15, 43.45)	-23.61 (-81.42, 34.20)
One-month follow-up T2	290.28 ± 111.05	335.93 ± 180.39	
Within-group changed T2	23.15 (-16.71, 63.02)	55.87 (12.69, 99.06)	-45.64 (-110.25, 18.97)
*Left Frontal muscles*			
Baseline	292.64 ± 117.05	266.25 ± 115.80	
Post-intervention T1	262.41 ± 130.66	269.78 ± 164.12	
Within-group changed T1	-30.23 (-66.25, 5.79)	3.53 (-38.52, 45.57)	-7.36 (-73.93, 59.20)
One-month follow-up T2	286.90 ± 122.80	321.60 ± 152.53	
Within-group changed T2	-5.74 (-43.68, 31.19)	55.35 (13.66, 97.04)	-34.70 (-96.83, 27.43)
*Right Temporal muscles*			
Baseline	251.18 ±75.90	241.75 ± 85.26	
Post-intervention T1	224.38 ± 81.85	261.70 ± 99.82	
Within-group changed T1	-26.79 (-55.20, 1.61)	19.95 (-6.11, 46.01)	-37.31 (-78.27, 3.64)
One-month follow-up T2	256.95 ± 77.07	289.90 ± 104.62	
Within-group changed T2	5.77 (-19.65, 31.19)	48.15 (11–02, 85.28)	32.95 (-74.20, 8.30)
*Left Temporal muscles*			
Baseline	266.92 ± 89.07	253.65 ± 99.55	
Post-intervention T1	224.33 ± 84.31	258.00 ±108.17	
Within-group changed T1	-42.59 (-78.18, -6.99)	4.35 (-23.09, 31.79)	-33.67 (-77.19, 9.86)
One-month follow-up T2	239.41 ± 69.48	293.50 ± 143.53	
Within-group changed T2	-27.51 (-59.16, 4.13)	39.85 (-8.44, 88.14)	-54.09 (-104.82, -3.36)
*Right Parietal suture*			
Baseline	294.79 ± 113.39	301.80 ± 141.72	
Post-intervention T1	268.31 ± 143.51	311.72 ± 156.52	
Within-group changed T1	-26.49 (-80.51, 27.53)	9.92 (-28.44, 48.29)	-43.42 (-110.74, 23.91)
One-month follow-up T2	286.33 ± 87.56	354.15 ± 141.46	
Within-group changed T2	-8.46 (-50.86, 33.94)	52.35 (-93.35, -11.35)	-67.82 (-120.68, .14.95)
*Left Parietal suture*			
Baseline	276.59 ± 108.79	280.23 ± 121.26	
Post-intervention T1	267.18 ± 110.55	290.65 ± 138.23	
Within-group changed T1	-9.41 (-44.75,25.93)	10.43 (-32.40, 53.25)	-23.47 (-79.63, 32.69)
One-month follow-up T2	263.82 ± 90.88	316.30 ± 130.83	
Within-group changed T2	-12.77 (-55.59, 30.05)	36.07 (-7.90, 80.05)	-52.48 (-103.07, -1.89)

The GROC-scale showed a clinical improvement in 77.5% of the subjects in the intervention group and 2.6% of the participants in the control group at T1 follow-up (*p* < 0.001). In T2 follow-up, 77.5% of the participants felt clinically better in the intervention group and 5.1% in the control group (*p* < 0.001).

## Discussion

This randomized controlled trial aimed to evaluate the effectiveness of three treatment sessions of diacutaneous fibrolysis in headache frequency, pain intensity, cervical pressure pain thresholds and cervical mobility in patients with tension-type headache.

The subjects who received the intervention obtained an improvement in the frequency of headache episodes, which is more remarkable considering that the control group worsened compared to the episodes suffered in the same period.

An improvement in the intensity of the headache was obtained for the four recorded situations, this reduction being in favor of the intervention group. Furthermore, cervical range of motion improved and the sensibility of the assessed pericranial tissue decreased.

In a recent review and meta-analysis, Kamonseki et al. [15] have found that other techniques of manual therapy such as soft tissue treatment and dry needling have been shown to be superior to no treatment on frequency of pain in tension-type headache. Soft tissue treatment techniques have also been shown to be more beneficial than other types of treatment techniques, but with less difference.

The intervention group’s frequency decreased significantly in 7.45 days (-10.38, -4.52), while the control group showed an increase of 2.31 days. It seems that the treatment of the musculature applied in the cranio-cervical region is an effective strategy to reduce the frequency of headache in subjects with tension-type headache. Other authors collect the frequency of headache suffered in two weeks or consider a specific percentage reduction in episodes to classify them as having improved [33]. Heterogeneity in the collection of frequency makes it difficult to compare results between studies.

After applying the intervention protocol, a decrease in the usual headache, and the worst headache felt in the last 4 weeks was observed, the decrease was 1.76 for the usual headache and 1.81 for the worst headache. Manual therapy has been suggested to be effective in reducing headache intensity in this type of subjects. Different authors have obtained changes of 1 to 3.1 cm in this scale with manual therapy treatment. These studies were carried out between eight and twenty treatment sessions, compared to the three applied in our study [24, 34, 35]. The study carried out by Toro Velasco et al. [36] would be the treatment that most closely resembles ours in terms of the dose and treatment goal.

Two suboccipital massage sessions were performed, obtaining an immediate improvement of 1 cm in the visual analogue scale. Our treatment achieved improvements superior to this but with a slightly higher dose.

Pericranial tenderness is one of the common symptoms in patients with tension-type headache. The pressure pain threshold is one of the most common ways of objectifying pain in mechanosensitive points. In the review published by Fernández de las Peñas et al. [6], it is observed that variables as the gender or the type of tension-type can affect the pressure pain thresholds, sensitivity to pressure pain was widespread only in chronic tension-type headache and women had lower pressure than men. Ferragut Garcías et al. [37] carried out a protocol of soft-tissue techniques and neural mobilization evaluating the effect on the pressure pain thresholds on two temporal points and the supraorbital region. After six treatment sessions, the neural mobilization group and the soft-tissue group obtained improvements in pressure pain thresholds, achieving an increase of 41.7% to 67.5% in the pressure pain thresholds’ values compared with the baseline. In this study, an increase in mechanosensitivity to pressure was observed in the control group for all the points assessed both between the baseline and T1 and between the baseline and T2.

On the other hand, we observed a decrease in mechanosensitivity for all the points evaluated in the intervention group except for the trapeziometacarpal joint between baseline and T1. As points to be highlighted, it is observed that in the intervention group between the baseline and T2, the mechanosensitivity to pressure had increased by 15.7% for the left temporal muscle, by 19.95% in the right frontal muscle, by 20.78% in the left frontal muscle and by 17.34% on the right parietal. For these points, a change greater than 15% was obtained concerning baseline for temporal muscle, frontal muscle, and right parietal suture. This percentage of change is considered the minimum clinically relevant change for pressure pain thresholds [38]. After local treatment, this widespread hypoalgesic response has been suggested to be produced by activating supraspinal pain inhibitory mechanisms, which are activated by mechanical force from manual therapy interventions [39].

It should be noted that for all movements, the intervention group improved range of motion. However, the control group maintains the range of cervical motion or even in some cases, experiences a reduction. Despite these trends, in most cases the minimum change detectable value is not reached, calculated with the CROM tool for subjects with cervical pain due to Fletcher et al. [40].

Considering these promising findings, it could be recommended the use of diacutaneous fibrolysis treatment in tension-type headache. It seems that the three treatments with this type of therapy reduces the headache pain intensity after the intervention protocol and is maintained at one-month follow-up (T2). It also improves the cervical and upper cervical range of motion in all cervical movements. The pressure pain thresholds increase therefore, it seems that the local and distal hyperalgesia decrease.

The present study has some limitations that need to be addressed. Our sample included subjects with medical diagnosis of tension-type headache without differentiating between frequent episodic or chronic type. It is possible that the differentiation between these subclassifications may be important in terms of the improvement of each type of patient [11]. Another limitation of this study has been the impossibility to blind the physiotherapist with respect to the applied interventions. There is another limitation regarding the follow-up, which may have been brief if considering that some subjects suffered from chronic tension-type headache. Finally, the medication consumed by the patients were no registered at T1 and T2 follow-ups, the change in consumption of the medication could be a confounding variable that could benefit or harm the results of this study.

For future studies, it would be advisable to extend the follow-up period to analyze the long-term effects that treatment with diacutaneous fibrolysis may have, as well as to associate it with soft tissue self-treatment techniques that can maximize the effect obtained.

## Conclusions

Diacutaneous fibrolysis is effective to improve short-term and one month follow-up headache frequency and intensity and provides improvements in range of motion in patients with tension-type headache.

The application of three sessions of diacutaneous fibrolysis in the cervico-dorsal and the orofacial areas in patients with tension-type headache produced a self-perceived improvement compared to control subjects.

## Supporting information

S1 File Study protocol in English. (PDF)

S2 File Study protocol in Spanish. (PDF)

S1 Checklist CONSORT 2010 checklist of information to include when reporting a randomised trial*. (DOC)

S1 Data https://doi.org/10.7910/DVN/MFXI4V. (TXT)
