# Supplementary material for: Effects of diacutaneous fibrolysis in patients with tension-type headache: A randomized controlled trial
Source: PLoS One. 2023 Mar 27;18(3):e0273877. doi: 10.1371/journal.pone.0273877 (PMC10042356; doi:10.1371/journal.pone.0273877)
Supplement: S1 File — (PDF) [file pone.0273877.s001.pdf]

COMITÉ ETICO DE INVESTIGACION CLINICA DE ARAGON

Avda. San Juan Bosco, 13
50009 Zaragoza

STRUCTURE OF A RESEARCH PROTOCOL AND RECOMMENDATIONS FOR ITS DEVELOPMENT

Tittle

Diacutaneous Fibrolysis, effects on Tension-type Headache

Research team

Thesis Director: Jose Miguel Tricás Moreno

Principal investigator: Sara Cabanillas Barea

Researchers: Andoni Carrasco Uribarren

Miguel Malo Urriés

Jaime Estéban Pérez

Center where the study was carried out: FCS Universidad de Zaragoza

Project

THEORETICAL-CONCEPTUAL CONTEXT

INTRODUCTION. TENSION-TYPE HEADACHE.
DEFINITION

Tension-type headache is a type of primary headache that was first described in 1988 by the International Headache Society. Several types of headaches with common clinical features are included within the term tension headache. The latest edition of the 2013 International Classification of Headaches (ICHD-III), includes the following main characteristics for this type of headache: pain in the cephalic region typically bilateral, of oppressive quality, with mild to moderate intensity, with a duration ranging from minutes to days. The pain does not worsen with physical activity and is not associated with nausea, photophobia, or phonophobia (but may occasionally be present).

If the duration of the episodes and their frequency are taken into account, four subtypes are established as shown in the following classification:

1. Infrequent Episodic.
    - 1.1. Associated with pericranial tenderness.
    - 1.2. Not Associated with pericranial tenderness.
2. Frequent Episodic.
    - 2.1. Associated with pericranial tenderness.
    - 2.2 Not Associated with pericranial tenderness.
3. Chronic.
    - 3.1 Associated with pericranial tenderness.
    - 3.2 Not Associated with pericranial tenderness.
4. Probable.
    - 4.1. Infrequent.
    - 4.2. Frequent.
    - 4.3. Chronic.

EPIDEMIOLOGICAL DATA OF TENSION-TYPE HEADACHE
The prevalence of tension-type headache remains stable throughout the patient's life, reaching its maximum value of intensity in the 30s (1–3) for, from the age of 70, progressively reduce (4). It is more common in women than in men, with a ratio between 5:4 and 3:2 (5,6).

PREVALENCE

Prevalence data for this type of headache are quite heterogeneous, varying between 20% and up to 70% depending on the clinical criteria used, the characteristics of the sample and the geographical area in which the data are collected or the year in which they are collected and which the studies are carried out.

Worldwide, data collected in primary care services indicate that the form of episodic tension-type headache affects more than 80% of the population at some point in life, and 10% of this population recurs frequently. For chronic tension headache, the data range around 2-3% in adult subjects (7).

In the latest edition of the International Classification of Headaches (ICHD-III), they point out that the data vary between 30% and 78%, they are the highest prevalence figures among all the types of headaches described (8) .

It seems that the prevalence of this type of headache is increasing in recent years (9). According to the Global Burden Disease of 2010, tension-type headache along with migraine appear as the second and third most prevalent dysfunction worldwide (10). North America in the first place and Europe in second, are the continents where this dysfunction is most detected (11).

INCIDENCE

The incidence of tension-type headache considered as new-onset cases seems difficult to quantify. The data vary between 14 to 44 cases per 1000 people per year for tensión-type headache (3,12).

DATA IN SPAIN

In 2008, in a hospital in the province of Valladolid, the characteristics of the first 1,000 cases that attended consultations for headaches were analyzed. Following the ICHD-II criteria, 51.4% of cases were classified as migraine, 16% tension-type headache, 3.4% cranial neuralgia, and 2.4% trigeminal autonomic headache. The female/male ratio was 2.46/1. And the mean age was 43.19 years $\pm$ 17.1, coinciding with data from other population studies (13).

Another older study carried out in Aragón in 1995 analyzed the characteristics of the patients who attended the neurology service in the community, and of these, which presented headaches. During 3 months, 3,489 patients attended the neurology service, of which 25.5% requested consultation due to headaches. 42% diagnosed with migraines and 30.1% with chronic tension-type headache (14). If we compare the data of these two studies with others carried out in the general population, we can think that

it would be necessary to take into account that migraine is more frequent in specialty consultations while tension-type headache is considered more prevalent in the general population and in consultations of Primary Care.

Socioeconomic effects

Tension headache can cause high levels of disability, not only to patients and their families, but also to society due to its high prevalence (11). In a study carried out in several European countries during the years 2008 and 2009, they calculated that the socioeconomic cost produced by patients with tension-type headache between 18 and 65 years old amounted to 21 billion euros, which corresponded to an average per patient of 303 euros per year. year (15).

CLINICAL PRESENTATION

In addition to the common clinical features described in the definition, tension headache subtypes are mainly classified into chronic and episodic depending on how the frequency and duration of symptoms vary.

The most frequent common clinical findings in these patients are increased sensitivity to manual palpation in the pericranial myofascial structures. (16–19) and the increase in the tone of the muscles of the cervical, cranial and scapular region (20). The tone of the craniocervical-scapular muscles and local sensitivity are closely related, without presenting a correlation with the periods of headache. This suggests that this musculature remains constantly altered and is not dependent on episodes of pain (21).

Another symptom that is frequently associated with tension headache is cervical pain, which appears in up to 88.4% of these patients (22).

On many occasions it is common to find myofascial structures with painful points on palpation (trigger points), decreased pressure pain threshold or postural alterations such as head thrust (20). Symptomatic alterations of the myofascial structures defined as active trigger points are prevalent in both adults and children in muscles such as the upper trapezius, the sternocleidomastoid or the temporal (23). For the suboccipital musculature, a typical area of referred pain is defined over the occipital and towards the temporal area, which is frequently perceived as bilateral headache. In patients with episodic tension-type headache, active trigger points have been found in 60% of cases, and in the remaining 40% they appeared in a latent state (24). In the upper trapezius, an increase in pain sensitivity has also been evidenced, both in the skin and in the muscle, compared to distal regions in the lower extremity for the same patients with chronic tension-type headache. (25).

ETIOLOGY AND RISK FACTORS

Some of the risk factors that favor the development of tension-type headache have been described: low socioeconomic status, poor self-perceived health status, difficulty relaxing after work, frequent use of medications or sleeping few hours per night are associated with appearance and perpetuation of this type of headache (12,26).

PATHOPHYSIOLOGY

The causal mechanisms of tension headache are not fully understood. Although it seems that in the frequent and infrequent episodic mode peripheral mechanisms may be more involved, in the chronic mode more central pain mechanisms are considered as the cause of the symptoms (8). Currently, despite having diagnostic criteria clearly defined by the ICHD, tension-type headache is not a perfectly defined entity in terms of its clinical and pathophysiological aspects.

Painful stimuli from sensitized pericranial tissues can cause referred pain to the head and be perceived as headache. Specifically, the myofascial trigger points of the muscles of the cervical and scapular area may be involved in the genesis of tension headache.

It seems that the dysfunction of peripheral mechanisms is more important in the type of episodic tension-type headache (27–29) and yet, the process of central sensitization in many cases is the mechanism that causes the headache to become chronic (30). This central sensitization process has been related both to nociceptive stimuli maintained over time (due to the release of algogenic substances that stimulate peripheral nociceptor zones) and to dysfunction of the descending inhibition system (28,29).

Some of these neurotransmitter and neuromodulator substances (nitric oxide, peptide related to the calcitonin gene, substance P...) are involved in the chronicity of pain in patients with tension headache (19). These algogenic substances that contribute to peripheral sensitization appear in in vivo analysis of active trigger points (those points whose pattern of referred pain is responsible for the patient's symptoms) and do not appear in latent trigger points or in control points (31).

All this information makes us think that alterations in the sensitive mechanisms of muscle, tendinous and fascial tissues of the cranial and cervico-scapular region may play an important role in the genesis of tension headache. But currently, the lack of knowledge of the pathophysiology of this headache affects the understanding of both the effectiveness of treatment techniques and the prevention of chronification.

DIAGNOSIS

The diagnosis of this type of headache is made based on the patient's clinical history and examination. Generally, the medical specialist establishes the diagnosis following the ICHD-3 diagnostic criteria.

If a patient meets the clinical criteria for tension-type headache and has normal neurological examination results, further testing is usually not necessary.

Manual palpation of the pericranial musculature is a very valuable but rarely used examination. Sensitivity of these muscles to palpation is the most common abnormal finding in tension-type headache, although its absence does not rule out tension-type headache. In some cases, it is useful to perform an eye fundus in order to rule out the presence of papilledema or other anomalies that may indicate the presence of secondary headaches (32).

TREATMENT

From primary care consultations, which constitute around 80% of the reference consultation points for these patients (33), the most used treatment is pharmacological, which usually consists of simple analgesics and NSAIDs for cases of episodic tension headache, also in some cases combinations of analgesics containing caffeine are prescribed. For cases of chronic tension headache, tricyclic antidepressants such as amitriptyline are sometimes prescribed (34,35).

Manual physiotherapy treatment and the use of acupuncture have shown beneficial effects for the relief of symptoms in patients with frequent tension-type headache despite the lack of solid evidence to support their use (35). The treatment techniques used in physiotherapy, both passive and active, have multiple physiological effects. Mainly, symptom relief in patients with headaches is explained by two mechanisms: reduction of sensitization of peripheral structures and activation of descending inhibitory pathways (28).

DIACUTANEOUS FIBROLYSIS

Diacutaneous fibrolysis (DF) is a treatment method used in the field of physiotherapy that aims to relieve mechanical pain in the musculoskeletal system. For this, "hooks" are used that, applied to the skin, destroy adhesions and irritative interaponeurotic or myofascial corpuscles, improving the mobility of the myofascial tissue in relation to all the adjacent structures. The term diacutaneous means "against the skin", as opposed to the term percutaneous which implies passing through the skin.

For the A.E.F.D (Spanish Association of Diacutaneous Fibrolysis) DF is defined as "specific instrumental intervention to normalize function in the musculoskeletal system, after an accurate diagnosis and preserving the integrity of the skin".(36)

In the 1960s, the Swedish physiotherapist Kurt Ekman began the development of this technique (37), being later modified by the Belgians Jean Burnotte and Pierre Duby (38). The reputation of this method was given due to the success in the treatment of occipitalgia, epicondylitis and rebellious tendinitis of the Achilles tendon.

This method implies a painless way of working, based on a centripetal approach towards the most symptomatic point in the tissue that can be treated. This treatment involves three successive phases to reduce the aggressiveness of the technique: digital palpation, instrumental palpation and fibrolysis. Digital palpation aims to locate intermuscular septa and anatomical structures of interest, instrumental palpation allows precise detection of points where the connective tissue has poorer mobility or adhesions between adjacent tissues are suspected, and fibrolysis is performed by additional traction with the hook designed to improve those adhered fibers and improve mobility between muscle compartments.

Initially Ekman and Colombo pointed to a purely mechanical effect of the technique, through the destruction of these adhesions and irritating corpuscles (37,39) while its successors also observed a positive effect on post-traumatic edema and symptoms of neurological pathologies (38).

This technique allows treating the myofascial tissue with more specificity, precision and depth than most of the techniques used in manual therapy for the treatment of tension headache.

To date, only four indexed works have been found that study the effect of this technique. All of them do it by applying fibrolysis in regions of the extremities, obtaining beneficial results on the pain and mobility of the joints on which it is applied (40–43).

PROJECT JUSTIFICATION

Tension-type headache is a very prevalent pathology in the general population that, despite the wide variety of treatment techniques available, in many patients presents persistent symptoms and a tendency to chronicity.

In my daily clinical experience with patients affected by tension headache, many of them state that DF is the technique that is achieving permanent benefits in the course of their symptoms. DF, by producing appreciable results from the first sessions, contributes not only to improving the patient's clinical situation, but also to increasing the results of the rest of the applied techniques and treatments.

The good clinical results of the DF, in this and in other pathologies, make it have a growing boom among physiotherapists, both nationally and internationally. However, the effectiveness of DF has not been proven in clinical trials on tension headache.

The aim of this clinical trial is to objectify the clinical improvements of these patients and to verify if DF really provides an additional advantage to the usual treatment of tension-type headache.

If its efficacy is confirmed, it would represent an advance in the treatment of patients affected by tension-type headache, a very prevalent and disabling pathology that, despite the multiple treatments available, continues to be a challenge for physiotherapists in daily clinical practice.

HYPOTHESIS AND OBJECTIVES

HYPOTHESIS

The diacutaneous fibrolysis technique on the myofascial structures of the cranio-cervical region in patients with tension-type headache produces a reduction in the frequency and intensity of pain and an improvement in the function of the cervical region, as well as in disability and quality of life, perceived by patients.

OBJECTIVES

MAIN OBJECTIVE

To identify the clinical results produced by an intervention by means of diacutaneous fibrolysis on the musculature of the cervical spine in patients with tension-type headache.

SECONDARY OBJECTIVES

- To describe the clinical effects produced by a diacutaneous fibrolysis intervention on myofascial structures of the craniocervical region in different aspects of pain in patients with tension headache.
- To describe the clinical effects produced by an intervention by means of diacutaneous fibrolysis on myofascial structures of the cranio-cervical region in the joint and muscular function of the cervical region in patients with tension-type headache.

- To describe the clinical effects produced by a diacutaneous fibrolysis intervention on myofascial structures of the cranio-cervical region in the posture of patients with tension-type headache.
- To describe the clinical effects produced by a diacutaneous fibrolysis intervention on myofascial structures of the craniocervical region on disability in patients with tension headache.
- To describe the clinical effects produced by a diacutaneous fibrolysis intervention on myofascial structures of the cranio-cervical region on quality of life in patients with tension headache.
- To relate the aforementioned clinical effects with the clinical characteristics of patients with tension-type headache, with the aim of establishing clinical prediction rules for the diacutaneous fibrolysis technique in this subgroup of patients.

METHODOLOGY

DESIGN

To achieve the objectives of the study, a randomized controlled clinical trial will be designed to evaluate the clinical effects of the application of diacutaneous fibrolysis compared to the usual conservative treatment. Given its characteristics, it will be an analytical, longitudinal, prospective and experimental study.

ETHICAL ASPECTS

This project will be presented to the Clinical Research Ethics Committee of Aragon (CEICA) and its approval is necessary to continue with the following stages of the study.

STUDY VARIABLES

INDEPENDENT VARIABLES

- Interventional treatment by diacutaneous fibrolysis on myofascial structures of the cranio-cervical region in the prone position.

- Control group, which will remain in the supine position for the same time as the intervention group with similar environmental conditions.

VARIABLE	TYPE	TOOL	VALUE
Age	Discrete quantitative	Anamnesis	Years
Gender	Nominal qualitative		Man / Woman
BMI	Discrete quantitative		Km/m^2
Toxic habits	Nominal qualitative		alcohol / tobacco / drugs
Work activity	Nominal qualitative		Active / Unemployed / Retired
Position work activity	Nominal qualitative		Seated / Standing / Ambulation / Combined
Hours of work activity	Discrete quantitative		Hours
Physical activity	Nominal qualitative		Yes / No
Hours of physical activity	Discrete quantitative		Hours
Analgesic medication	Nominal qualitative		Yes / No
Headache history	Continuous quantitative		Years
headache frequency	Discrete quantitative		Days
Headache duration	Discrete quantitative		Minutes
Location	Nominal qualitative		Frontal / Occipital / Temporal / Parietal
Quality of pain	Nominal qualitative		Oppressive / Throbbing / Continuous
Pain intensity	Discrete quantitative		VAS (100mm)
Associated signs	Nominal qualitative		Vomiting / Phonophobia / Photophobia / Others / None
Need to lie down	Nominal qualitative		Yes / No
Triggers	Nominal qualitative		Yes / No
Aggravating factors	Nominal qualitative		Yes / No

DEPENDENT VARIABLES

VARIABLE	TOOL
Pain intensity	Visual analogue scale (r=0.94, P < 0.001) (44)
Frecuency	Registration sheet
Duration	Registration sheet
Localization	Registration sheet (topographic map)
Pressure pain threshold	Pressure algometer r=0,92 (ICC) (45)
Muscle tenderness	Manual assessment (criteria according to Langemark and Olesen)(18)
Mobility of the intermuscular septa	Manual asessment
Muscle length	Manual asessment
Deep flexor muscle strength	Cranio-cervical flexion test r=0.63-0.86 (ICC) (46)
Cervical Range of Motion	CROM r=0.87-0.94 (ICC) (46)
Forward head position	Photographic camera interexaminer r>0.85(47)
Cross section of cervical muscles	Ultrasound
Disability	HIT-6 Questionnaire
Quality of life	COOP-WONCA Questionnaire

The variables have been chosen based on a review carried out in 2013 by J. Abboud in which he selected the most used musculoskeletal variables in the evaluation of patients with tension-type headache in clinical trials. (20). In addition, variables have been added that assess muscle parameters that may be relevant, as well as quality of life and disability to see the impact of this pathology on the daily life of these patients.

STUDY SAMPLE

SAMPLE SIZE

The variable improvement in headache frequency has been chosen as a reference for calculating the sample size.

The calculation was made with the online program GRANMO 7.12 with the comparison menu between two independent means with a unilateral contrast and the following values: alpha risk 0.05; beta risk 0.20; to detect a difference equal to or greater than 3 units; a common standard deviation of 5 units; a ratio between the number of subjects between groups 1 and estimating a 15% loss to follow-up. The result is that 41 subjects per group are needed, as in this study there will be two groups, there are 82 subjects in total. Recruitment of this number of subjects is expected to be feasible.

The statistical data necessary to perform the calculation have been obtained from the study by Castien et al.(48) on the comparison of the application of manual therapy techniques versus usual medical treatment in patients with chronic tension-type headache, with an outpatient population in the Netherlands area.

Recruitment and obtaining the sample

Participation in the study will be offered to patients from different primary care services in the Zaragoza metropolitan area. Those patients who, being diagnosed with tension-type headache by the doctor responsible for each service, may be likely to benefit from participation in this study will be informed of the study.

INCLUSION AND EXCLUSION CRITERIA

Inclusion criteria:

- Present a diagnosis of episodic or chronic tension-type headache, made by a medical professional in the area of neurology, following the fundamental diagnostic criteria for tension-type headache established in the International Classification of Headaches (ICHD-III).
- Be over 18 years of age.
- Ability to fill in the questionnaires.
- Attendance at all intervention and assessment sessions.
- Reading of the informative document and signing of the informed consent.

Exclusion criteria:

- Involvement in compensation or litigation for health problems.
- Receive physiotherapy treatment in the cranial-cervical region in the month prior to the study or during it. However, the subject is allowed to continue on palliative medication that continues until the completion of the study for ethical reasons and to facilitate the accuracy of the study.
- Subjects with the presence of red flags for headaches(49), as well as subjects who may have severe diseases that may be related to clinical outcomes: malignancy or history of cancer, vertebral infection, tumors or vertebral fractures, lumbar instability, blood dyscrasia, severe trauma in the previous 3 months, neck surgery in the previous 12 months, referred neck pain of visceral origin.
- Insufficient understanding of Spanish.
- Change of usual medication guidelines in the last month.
- Not completing all the proposed sessions.

RANDOMIZATION

The subjects will be randomly assigned to an Intervention Group by means of FD in the cranio-cervical region (Group A) or to a Control Group (Group B). The randomization process will be carried out through a systematic random sampling, together with the creation of a list of random numbers (1 and 2) created from a computer application for randomization of numbers (1 = Group A; 2 = Group B).

EDSCRIPTION OF THE INTERVENTION

Once the sample has been selected, an initial evaluation will be carried out, collecting data on the patient's clinical history using the anamnesis variables mentioned above. In addition, to identify more clinical characteristics of the sample of patients with tension-type headache, together with the clinical history, an assessment will be made of: the pressure pain threshold in the most relevant muscles of the cervical region by assessment with an algometer of pressure, cervical range of motion in the three planes of space using the CROM cervical range of motion measuring device and muscle length of the cervico-scapular muscles, the forward head position in sitting and standing by photometry, the resistance of the flexor musculature by means of the craniocervical flexion test (50) and the cross-sectional area of the deep cervical musculature by ultrasound.

Both groups will attend 3 sessions on alternate days for a week. Each of the sessions will consist of a pre-intervention assessment (intensity, frequency and location of the pain), an intervention (DF in the cranio-cervical region / Control) and a post-intervention assessment (intensity, frequency and location of the pain). pain). In addition, a final complete assessment will be carried out 4 weeks after the last intervention. This assessment will be carried out throughout the study in the same time slot and will be preceded by some active warm-up exercises by the patient. The approximate time of each session will be 60 minutes.

Each treatment session of the Intervention Group will consist of a DF session in the cranio-cervical region, for 30 minutes, which will be directed mainly to the approach to the edges, insertions and insertional tendinous tissue of the following muscles: trapezius, paravertebral, angular scapulae, splenius capitis and neck, suboccipital, sternocleidomastoid, and temporal. The patient will be informed that the technique must be painless at all times and only a slight pricking sensation is allowed during hook traction (below 3 out of 10 on a VAS scale). The Control Group will receive the same assessments and will remain in the supine position for the same treatment time without receiving any intervention.

Participants in both groups will be asked not to modify treatment guidelines or regular physical exercise, or hours of sleep and, to the extent possible, not to vary very markedly the activities of their daily life during the period. how long the study lasts.

DATA COLLECTION AND ANALYSIS

Those patients of the previously mentioned medical services, likely to participate in this study according to the corresponding medical specialist and who meet the inclusion and exclusion criteria will be referred to a physiotherapist to provide them with the information (oral and written) of the study. Those patients who decide to collaborate must sign the informed consent and will be assigned a correlative number. This same physiotherapist will be in charge of carrying out the initial, post-intervention and follow-up assessment, remaining blind to the assigned group. This assessment will include: Anamnesis, distribution of questionnaires, collection of data on the examination sheet related to pain, examination of the joint and muscular function of the cervical region, taking photographs to analyze posture and performing several ultrasounds of the cervical musculature.

A second physiotherapist, who will be the only one with access to the randomization list, will apply the diacutaneous fibrolysis technique to the patients assigned to the intervention group. It will also record the possible incidents or complications that may have occurred, such as bruises, skin reactions, etc.

As it is a manual technique, the physiotherapist cannot be blinded to the patient's condition, but allocation concealment is respected with this procedure.

STATISTIC ANALYSIS

The statistical study will be carried out with the SPSS version 22.0 for Mac program. The confidence level established for the analysis of the results will be 95%. The person who will analyze the results will be blinded to the interventions assigned to each subject.

To achieve the objectives of the study, a descriptive study, a comparative study, a correlational and regression study of the collected data will be carried out.

For the descriptive analysis of the quantitative variables, the central tendency indices (mean and median) and the dispersion indices (standard deviation and the minimum and maximum values) will be used. For qualitative variables, a study of frequencies will be carried out to find out the absolute and relative frequencies, and the valid and cumulative percentages. In some cases, the mode central tendency measure will be extracted.

Prior to carrying out the comparative study between both groups, a study will be carried out to determine the normality and homoscedasticity of the study's scale variables and thus be able to use the corresponding statistical operations.

WORKPLAN

STAGES OF DEVELOPMENT

1. Complete the literature search: January 2015
2. Analysis of the bibliography and drafting of the project: April 2015
3. Submission to the Ethics Committee: June 2015
4. Recruitment of subjects: September 2015 - September 2016
5. Data analysis: November 2016
6. Writing of the results: February 2017
7. Presentation of the thesis: Spring 2017

PLACE OF REALIZATION OF THE PROJECT

The study will be carried out in the facilities of the Faculty of Health Sciences of the University of Zaragoza.

LIST OF REFERENCES

1. Fernández-de-Las-Peñas C, Ge H-Y, Alonso-Blanco C, González-Iglesias J, Arendt-Nielsen L. Referred pain areas of active myofascial trigger points in head, neck, and shoulder muscles, in chronic tension type headache. J Bodyw Mov Ther. 2010 Oct;14(4):391–6.

2. Gemma V E-L, Antonia G-C. Efficacy of manual and manipulative therapy in the perception of pain and cervical motion in patients with tension-type headache: a randomized, controlled clinical trial. J Chiropr Med. 2014 Mar;13(1):4–13.

3. Ashina S, Bendtsen L, Ashina M. Pathophysiology of migraine and tension-type headache. Tech Reg Anesth Pain Manag. 2012;16:14–8.

4. Ferrante T, Manzoni GC, Russo M, Camarda C, Taga A, Veronesi L, et al. Prevalence of tension-type headache in adult general population: the PACE study and review of the literature. Neurol Sci. 2013 May;34 Suppl 1:S137–8.

5. Chowdhury D. Tension type headache. Ann Indian Acad Neurol. 2012 Aug;15(Suppl 1):S83–8.

6. WHO | Headache disorders [Internet]. World Health Organization; [cited 2015 May 19]. Available from: http://www.who.int/mediacentre/factsheets/fs277/en/

7. Steiner TJ, Martelletti P. Aids for management of common headache disorders in primary care. J Headache Pain. 2007 Oct;8 Suppl 1:S2.

8. The International Classification of Headache Disorders, 3rd edition (beta version). Cephalalgia. 2013 Jul;33(9):629–808.

9. Lyngberg AC, Rasmussen BK, Jørgensen T, Jensen R. Has the prevalence of migraine and tension-type headache changed over a 12-year period? A Danish population survey. Eur J Epidemiol. 2005 Jan;20(3):243–9.

10. Vos T, Flaxman AD, Naghavi M, Lozano R, Michaud C, Ezzati M, et al. Years lived with disability (YLDs) for 1160 sequelae of 289 diseases and injuries 1990-2010: A systematic analysis for the Global Burden of Disease Study 2010. Lancet. 2012;380(9859):2163–96.

11. Stovner L, Hagen K, Jensen R, Katsarava Z, Lipton R, Scher A, et al. The global burden of headache: a documentation of headache prevalence and disability worldwide. Cephalalgia. 2007 Mar;27(3):193–210.

12. Lyngberg AC, Rasmussen BK, Jørgensen T, Jensen R. Prognosis of migraine and tension-type headache: a population-based follow-up study. Neurology. 2005 Aug 23;65(4):580–5.

13. Guerrero ÁL, Rojo E, Herrero S, Neri MJ, Bautista L, Peñas ML, et al. Characteristics of the first 1000 headaches in an outpatient headache clinic registry. Headache. 2011 Feb;51(2):226–31.

14. Gracia-Naya M. [The importance of headaches in neurology clinics. Study groups of neurologists of Aragon]. Rev Neurol. 1999;29(5):393–6.

15. Linde M, Gustavsson A, Stovner LJ, Steiner TJ, Barré J, Katsarava Z, et al. The cost of headache disorders in Europe: the Eurolight project. Eur J Neurol. 2012 May;19(5):703–11.

16. Bendtsen L, Jensen R, Jensen NK, Olesen J. Pressure-controlled palpation: a new technique which increases the reliability of manual palpation. Cephalalgia. 1995 Jun;15(3):205–10.

17. Jensen R, Rasmussen BK, Pedersen B, Olesen J. Muscle tenderness and pressure pain thresholds in headache. A population study. Pain. 1993 Feb;52(2):193–9.

18. Langemark M, Olesen J. Pericranial tenderness in tension headache. A blind, controlled study. Cephalalgia. 1987 Dec;7(4):249–55.

19. Ashina M. Neurobiology of chronic tension-type headache. Cephalalgia. 2004 Mar;24(3):161–72.

20. Abboud J, Marchand A-A, Sorra K, Descarreaux M. Musculoskeletal physical outcome measures in individuals with tension-type headache: a scoping review. Cephalalgia. 2013 Dec;33(16):1319–36.

21. Ashina M, Bendtsen L, Jensen R, Sakai F, Olesen J. Muscle hardness in patients with chronic tension-type headache: relation to actual headache state. Pain. 1999 Feb;79(2-3):201–5.

22. Ashina S, Bendtsen L, Lyngberg AC, Lipton RB, Hajiyeva N, Jensen R. Prevalence of neck pain in migraine and tension-type headache: A population study. Cephalalgia. 2014 May 22;

23. Alonso-Blanco C, Fernández-de-las-Peñas C, Fernández-Mayoralas DM, De-la-Llave-Rincón AI, Pareja JA, Svensson P. Prevalence and anatomical localization of muscle referred pain from active trigger points in head and neck musculature in adults and children with chronic tension-type headache. Pain Med. 2011 Oct;12(10):1453–63.

24. Fernández-de-Las-Peñas C, Alonso-Blanco C, Cuadrado ML, Pareja J a. Myofascial trigger points in the suboccipital muscles in episodic tension-type headache. Man Ther. 2006 Aug;11(3):225–30.

25. Ashina S, Babenko L, Jensen R, Ashina M, Magerl W, Bendtsen L. Increased muscular and cutaneous pain sensitivity in cephalic region in patients with chronic tension-type headache. Eur J Neurol. 2005 Jul;12(7):543–9.

26. Katsarava Z, Dzagnidze A, Kukava M, Mirvelashvili E, Djibuti M, Janelidze M, et al. Primary headache disorders in the Republic of Georgia: prevalence and risk factors. Neurology. 2009 Nov 24;73(21):1796–803.

27. Jensen R, Bendtsen L, Olesen J. Muscular factors are of importance in tension-type headache. Headache. 1998 Jan;38(1):10–7.

28. Fernández-de-Las-Peñas C. Physical therapy and exercise in headache. Cephalalgia. 2008 Jul;28 Suppl 1:36–8.

29. Bendtsen L, Fernández-de-la-Peñas C. The role of muscles in tension-type headache. Curr Pain Headache Rep. 2011 Dec;15(6):451–8.

30. Bezov D, Ashina S, Jensen R, Bendtsen L. Pain perception studies in tension-type headache. Headache. 2011 Feb;51(2):262–71.

31. Shah JP, Phillips TM, Danoff J V, Gerber LH. An in vivo microanalytical technique for measuring the local biochemical milieu of human skeletal muscle. J Appl Physiol. 2005 Nov;99(5):1977–84.

32. Loder E, Rizzoli P. Tension-type headache. BMJ. 2008 Jan 12;336(7635):88–92.

33. Kristoffersen ES, Grande RB, Aaseth K, Lundqvist C, Russell MB. Management of primary chronic headache in the general population: the Akershus study of chronic headache. J Headache Pain. 2012 Mar;13(2):113–20.

34. Kaniecki RG. Tension-type headache. Continuum (Minneap Minn). 2012 Aug;18(4):823–34.

35. Bendtsen L, Evers S, Linde M, Mitsikostas DD, Sandrini G, Schoenen J. EFNS guideline on the treatment of tension-type headache - report of an EFNS task force. Eur J Neurol. 2010 Nov;17(11):1318–25.

36. Tricás-Moreno JM, Lucha-López O, Duby P. Fibrolisis diacutánea: según el concepto de Kurt Ekman. 1ª Edición. Asociación Española de Fibrolisis Diacutánea, editor. 2010.

37. Ekman K. Eine neue methode der fibrolyse zur unterstützung der manuellen therapie. Man Medizin. 1972;10:3–6.

38. Burnotte J, Duby P. Fibrolyse Diacutanée et algies de l'appareil locomoteur. Kinésithérapie Sci. 1988;271:16–8.

39. Colombo I, Ekman K. La fibrolisi diacutenea nuovo mezzo diagnostico e terapeutico in fisiatria. Eura Medicophys. 1968;4:29–36.

40. Barra ME, López C, Fernández G, Murillo E, Villar E, Raya L. The immediate effects of diacutaneous fibrolysis on pain and mobility in patients suffering from painful shoulder: a randomized placebo-controlled pilot study. Clin Rehabil. 2011;25(4):339–48.

41. Barra López ME, López de Celis C, Fernández Jentsch G, Raya de Cárdenas L, Lucha López MO, Tricás Moreno JM. Effectiveness of Diacutaneous Fibrolysis for the treatment of subacromial impingement syndrome: a randomised controlled trial. Man Ther. 2013 Oct;18(5):418–24.

42. Loro C, Lucha O, Caudevilla S, Marín E, Tricás JM, Estébanez E. Fibrolisis Diacutánea: tratamiento de un caso. Cuest Fisioter. 2000;14:9–15.

43. Tricás JM, Lucha O, García Bernabé. Tratamiento de un hombro congelado. Fundamentación teórica y caso clínico. Ter Man Venez. 1998;1:22–30.

44. Hawker GA, Mian S, Kendzerska T, French M. Measures of adult pain: Visual Analog Scale for Pain (VAS Pain), Numeric Rating Scale for Pain (NRS Pain), McGill Pain Questionnaire (MPQ), Short-Form McGill Pain Questionnaire (SF-MPQ), Chronic Pain Grade Scale (CPGS), Short Form-36 Bodily Pain Scale (SF. Arthritis Care Res (Hoboken). 2011 Nov;63 Suppl 1:S240–52.

45. Fischer AA. Pressure algometry over normal muscles. Standard values, validity and reproducibility of pressure threshold. Pain. 1987 Jul;30(1):115–26.

46. Jørgensen R, Ris I, Falla D, Juul-Kristensen B. Reliability, construct and discriminative validity of clinical testing in subjects with and without chronic neck pain. BMC Musculoskelet Disord. 2014 Dec 4;15(1):408.

47. Ruivo RM, Pezarat-Correia P, Carita AI. Intrarater and interrater reliability of photographic measurement of upper-body standing posture of adolescents. J Manipulative Physiol Ther. 2015 Jan;38(1):74–80.

48. Castien RF, van der Windt DAWM, Dekker J, Mutsaers B, Grooten A. Effectiveness of manual therapy compared to usual care by the general practitioner for chronic tension-type headache: design of a randomised clinical trial. BMC Musculoskelet Disord. 2009 Jan;10:21.

49. Fernández-de-las-Peñas C, Arendt-Nielsen L, Gerwin RD. Fisiopatología, diagnóstico y tratamiento. Cefalea Tensional y de Origen Cervical. Barcelona: Elsevier; 2010.

50. Jull G, Barrett C, Magee R, Ho P. Further clinical clarification of the muscle dysfunction in cervical headache. Cephalalgia. 1999 Apr;19(3):179–85.
