# Supplementary material for: Effects of diacutaneous fibrolysis in patients with tension-type headache: A randomized controlled trial
Source: PLoS One. 2023 Mar 27;18(3):e0273877. doi: 10.1371/journal.pone.0273877 (PMC10042356; doi:10.1371/journal.pone.0273877)
Supplement: S2 File — (PDF) [file pone.0273877.s002.pdf]

COMITÉ ETICO DE INVESTIGACION CLINICA DE ARAGON

Avda. San Juan Bosco, 13
50009 Zaragoza

ESTRUCTURA DE UN PROTOCOLO DE INVESTIGACIÓN Y RECOMENDACIONES PARA SU ELABORACIÓN

Título

Fibrolisis Diacutánea, efectos sobre la Cefalea Tensional

Equipo investigador

Director de tesis: Jose Miguel Tricás Moreno

Investigador principal: Sara Cabanillas Barea

Investigadores: Andoni Carrasco Uribarren

Miguel Malo Urriés

Jaime Estéban Pérez

Centro de realización del estudio: FCS Universidad de Zaragoza

Proyecto

CONTEXTO TEÓRICO-CONCEPTUAL

INTRODUCCIÓN. CEFALEA TENSIONAL.

DEFINICIÓN

La cefalea de tipo tensional es un tipo de cefalea primaria que fue descrita por primera vez en 1988 por la Sociedad Internacional de Cefaleas. Dentro del término cefalea tensional se incluyen varios tipos de cefaleas con características clínicas comunes. La última edición de la Clasificación Internacional de Cefaleas del 2013 (ICHD-III), recoge las siguientes características principales para este tipo de cefaleas: dolor en la región cefálica típicamente bilateral, de calidad opresiva, con una intensidad de leve a moderada, con una duración que varía de minutos a días . El dolor no empeora con la actividad física y no se asocia a náuseas, fotofobia o fonofobia (pero en ocasiones pueden estar presentes).

Si se tienen en cuenta la duración de los episodios y la frecuencia de estos, se establecen cuatro subtipos como muestra la siguiente clasificación:

1. Infrecuente Episódica.
    1.1. Asociada a sensibilidad pericraneal.
    1.2. No asociada a sensibilidad pericraneal.

2. Frecuente Episódica.
    2.1. Asociada a sensibilidad pericraneal.
    2.2 No asociada a sensibilidad pericraneal.
3. Crónica.
    3.1 Asociada a sensibilidad pericraneal.
    3.2 No asociada a sensibilidad pericraneal.
4. Probable.
    4.1. Infrecuente.
    4.2. Frecuente.
    4.3. Crónica.

DATOS EPIDEMIOLÓGICOS DE LA CEFALEA TENSIONAL

La prevalencia de la cefalea tensional se mantiene estable a lo largo de la vida del paciente, alcanzando su valor máximo de intensidad en la década de los 30 años (1–3) para, a partir de los 70 años disminuir progresivamente (4). Es más frecuente en mujeres que en hombres, oscilando el ratio entre 5:4 y 3:2 (5,6).

PREVALENCIA

Los datos de prevalencia para este tipo de cefalea son bastante heterogéneos, varían entre un 20% y hasta un 70% dependiendo los criterios clínicos utilizados, las características de la muestra y de la zona geográfica en la que se recogen los datos o del año en que se realizan los estudios.
A nivel mundial, datos recogidos en servicios de atención primaria, señalan que la forma de cefalea tensional episódica afecta a más de un 80% de la población en algún momento de la vida, y en un 10% de esta población recurre frecuentemente. Para la cefalea tensional crónica, los datos oscilan alrededor de un 2-3% en sujetos adultos (7).
En la última edición de la Clasificación Internacional de Cefaleas (ICHD-III), señalan que los datos varían entre el 30% y 78%, son las cifras más altas de prevalencia entre todos los tipos de cefaleas descritas (8) .
Sí que parece que la prevalencia para este tipo de cefalea está incrementando a lo largo de los último años (9). Según el Global Burden Disease de 2010, la cefalea tensional junto a la migraña aparecen como la segunda y la tercera disfunción más prevalente a nivel mundial (10). Norteamérica en primer lugar y Europa en segundo, son los continentes donde más se detecta esta disfunción (11).

INCIDENCIA

La incidencia de la cefalea tensional considerada como casos de nueva aparición, parece difícil de cuantificar. Los datos varían entre 14 a 44 casos por cada 1000 personas por año para cefalea tensional (3,12).

DATOS EN ESPAÑA

En el año 2008, en un hospital de la provincia de Valladolid se analizaron las características de los 1000 primeros casos que acudían a consultas por cefaleas. Siguiendo los criterios de la ICHD-II se clasificaron en migraña a un 51,4% de los casos, cefalea tensional a un 16%, neuralgias craneales 3,4% y cefaleas autonómicas trigeminales al 2,4%. El ratio mujer/hombre fue de 2,46/1. Y la media de edad fue de 43,19 años ± 17,1 coincidiendo con los datos de otros estudios poblacionales (13).
Otro estudio más antiguo realizado en Aragón en 1995, analizó las características de los pacientes que acudían al servicio de neurología en la comunidad, y de estos, cuales presentaban cefaleas. Durante 3 meses, acudieron al servicio de neurología 3489 pacientes, de los cuales un 25,5% solicitaron consulta por cefaleas. Un 42% diagnosticados de migrañas y un 30,1% de cefalea tensional de tipo crónica (14). Si comparamos los datos de estos dos estudios con otros realizados en población general, podemos pensar que sería necesario tener en cuenta que la migraña es más

frecuente en las consultas de especialidad mientras que la cefalea tensional se considera más prevalente en la población general y en consultas de Atención Primaria.

EFECTOS SOCIOECONÓMICOS

La cefalea tensional puede causar altos niveles de discapacidad, no sólo a los pacientes y a sus familias, sino también a la sociedad debido a su alta prevalencia (11). En un estudio realizado en varios países europeos durante los años 2008 y 2009, calcularon que es gasto socioeconómico producido por pacientes con cefalea tensional de entre 18 y 65 años ascendía a 21 billones de euros, que correspondía a una media por paciente de 303 euros al año (15).

PRESENTACIÓN CLÍNICA

Además de las características clínicas comunes descritas en la definición, los subtipos de cefaleas tensionales se clasifican principalmente en crónica y en episódica dependiendo de cómo varia la frecuencia y la duración de los síntomas.
Los hallazgos clínicos comunes más frecuentes en estos pacientes son el incremento de la sensibilidad a la palpación manual en las estructuras miofasciales pericraneales (16–19) y el aumento del tono de la musculatura de la región cervical, craneal y escapular (20). El tono de la musculatura cráneo-cervico-escapular y la sensibilidad local están íntimamente relacionados, sin presentar correlación con los periodos de cefalea. Esto sugiere que esta musculatura permanece constantemente alterada y no es dependiente de los episodios de dolor (21). Otro síntoma que con frecuencia se asocia a la cefalea tensional, es el dolor cervical, que aparece hasta en el 88,4% de estos pacientes (22).
En muchas ocasiones es frecuente encontrar estructuras miofasciales con puntos dolorosos a la palpación (puntos gatillo), disminución del umbral de dolor a la presión o alteraciones posturales como antepulsión cefálica (20). Las alteraciones sintomáticas de las estructuras miofasciales definidas como puntos gatillo activos, son prevalentes tanto en adultos como en niños en músculos como el trapecio superior, el esternocleidomastoideo o el temporal (23). Para la musculatura suboccipital se define una zona típica de dolor referido sobre el occipital y hacia la zona temporal, lo que se percibe frecuentemente como cefalea bilateral. En pacientes con cefalea tensional de tipo episódica, se han encontrado puntos gatillo activos en el 60% de los caso, y en el 40% restante aparecían en estado latente (24). En trapecio superior también se ha evidenciado incremento en la sensibilidad dolorosa tanto en la piel como en el músculo comparado con regiones distales en extremidad inferior para mismos pacientes con cefalea tensional de tipo crónica (25).

ETIOLOGÍA Y FACTORES DE RIESGO

Se han descrito algunos de los factores de riesgo que favorecen el desarrollo de cefalea tensional, un bajo estatus socioeconómico, bajo estado de salud autopercibido, dificultad para relajarse después del trabajo, el uso frecuente de medicamentos o dormir pocas horas por noche están asociados con la aparición y perpetuación de este tipo de cefalea (12,26).

PATOFISIOLOGÍA

Los mecanismos causales de la cefalea tensional no se conocen con exactitud. Aunque parece que en el modo frecuente e infrecuente episódico pueden estar más involucrados mecanismos periféricos, en el modo crónico se barajan más mecanismos de dolor central como causantes de los síntomas (8). Actualmente, la cefalea tensional a pesar de tener criterios diagnósticos claramente definidos por la ICHD, no es una entidad propia perfectamente perfilada en cuanto a sus aspectos clínicos y fisiopatológicos.
Los estímulos dolorosos de los tejidos pericraneales sensibilizados, pueden provocar dolor referido a la cabeza y ser percibido como cefalea. En concreto, los puntos gatillo

miofasciales de los músculos de la zona cervical y escapular pueden estar implicados en la génesis de la cefalea tensional.
Parece ser que la disfunción de mecanismos periféricos cobra más importancia en el tipo de cefalea tensional episódica (27–29) y sin embargo, el proceso de sensibilización central en muchos casos es el mecanismo que provoca que la cefalea se cronifique (30). Este proceso de sensibilización central se ha relacionado tanto con estímulos nociceptivos mantenidos a los largo del tiempo (debido a la liberación de sustancias algógenas que estimulan zonas de nociceptores periféricos) como con disfunción del sistema de inhibición descendente (28,29).
Algunas de estas sustancias neurotransmisoras y neuromoduladoras (oxido nítrico, péptido relacionado con el gen de la calcitonina, sustancia P…) están involucradas en la cronificación del dolor de los pacientes con cefalea tensional (19). Estas sustancias algógenas que contribuyen a la sensibilización periférica, aparecen en análisis invivo de puntos gatillo activo (aquellos puntos cuyo patrón de dolor referido es responsable de los síntomas del paciente) y no aparecen en puntos gatillo latentes o en puntos control (31).
Toda esta información, nos hace pensar que las alteraciones de los mecanismos sensitivos de tejidos musculares, tendinosos y fasciales de la región craneal y cervico-escapular pueden jugar un papel importante en la génesis de la cefalea tensional. Pero actualmente, el desconocimiento de la patofisiología de esta cefalea afecta a la comprensión tanto de la efectividad de las técnicas de tratamiento como al de la prevención de la cronificación.

DIAGNÓSTICO

El diagnóstico de este tipo de cefalea se realiza en base a la historia clínica del paciente y la exploración. Generalmente, el especialista médico establece el diagnóstico siguiendo los criterios diagnósticos de la ICHD-3.
Si un paciente cumple los criterios clínicos para cefalea tensional y tiene resultados normales en el examen neurológico, generalmente no es necesaria la realización de pruebas complementarias.
La palpación manual de la musculatura pericraneal es una exploración muy valiosa pero poco utilizada, la sensibilidad de estos músculos a la palpación es el hallazgo anormal más común en la cefalea tensional, aunque su ausencia no descarte la cefalea tensional. En algunos casos es útil realizar un fondo de ojo con el objetivo de descartar la presencia de un papiledema u otras anomalías que puedan indicar presencia de cefaleas secundarias (32).

TRATAMIENTO

Desde las consultas de atención primaria (que constituyen alrededor del 80% de los puntos de consulta de referencia para estos pacientes (33))el tratamiento más utilizado es el farmacológico, el cual consta habitualmente de analgésicos simples y AINES para los casos de cefalea tensional episódica, también en algunos casos se prescriben combinaciones de analgésicos que contengan cafeína. Para casos de cefalea tensional crónica, en ocasiones se prescriben antidepresivos tricíclicos como la amitriptilina (34,35).

El tratamiento fisioterápico manual y el uso de acupuntura han mostrado efectos beneficiosos para el alivio de síntomas en pacientes con cefalea tensional frecuente a pesar de no existir evidencia sólida que apoye su utilización (35). Las técnicas de tratamiento utilizadas en fisioterapia , tanto pasivas como activas, tienen múltiples efectos fisiológicos. Principalmente, el alivio de síntomas en pacientes con cefaleas se explica mediante dos mecanismos: reducción de la sensibilización de estructuras periféricas y activación de vías inhibitorias descendentes (28).

FIBROLISIS DIACUTÁNEA

La fibrolisis diacutánea (FD) es un método de tratamiento utilizado en el ámbito de la fisioterapia que tiene como objetivo el alivio de las algias mecánicas del aparato locomotor. Para ello se utilizan unos "ganchos" que aplicados sobre la piel destruyen adherencias y corpúsculos irritativos interaponeuróticos o mioaponeuróticos mejorando la movilidad del tejido miofascial en relación a todas las estructuras adyacentes. El término diacutánea significa "contra la piel", frente al término percutánea que implica atravesar la piel.
Para la A.E.F.D (Asociación Española de Fibrolisis Diacutánea) la FD se define como "intervención específica instrumental para normalizar la función en el sistema musculoesquelético, tras un diagnóstico preciso y conservando la integridad de la piel".(36)
En los años 60 el fisioterapeuta sueco Kurt Ekman inició el desarrollo de esta técnica (37), siendo modificada posteriormente por los belgas Jean Burnotte y Pierre Duby (38). La reputación de este método se dio debido al éxito en los tratamientos de occipitalgias, epicondilitis y tendinitis rebeldes del tendón de Aquiles.
Este método implica una forma de trabajar indolora, basándose en un abordaje centrípeto hacia el punto más sintomático en el tejido susceptible de tratamiento. Este tratamiento comporta tres fases sucesivas para disminuir la agresividad de la técnica: palpación digital, palpación instrumental y fibrolisis. La palpación digital tiene como objetivo localizar tabiques intermusculares y estructuras anatómicas de interés, la palpación instrumental permite detectar con precisión los puntos donde el tejido conjuntivo tiene peor movilidad o se sospecha de adherencias entre tejidos adyacentes y la fibrolisis se realiza mediante una tracción suplementaria con el gancho destinada a mejorar esas fibras adheridas y mejorar la movilidad entre compartimentos musculares.
Inicialmente Ekman y Colombo apuntaban a un efecto de la técnica puramente mecánico, mediante la destrucción de estas adherencias y corpúsculos irritativos (37,39) mientras que sus sucesores también observaron un efecto positivo sobre el edema postraumático y en los síntomas de patologías neurológicas (38).
Esta técnica permite tratar el tejido miofascial con más especificidad, precisión y a más profundidad que la mayoría de técnicas utilizadas en terapia manual para el tratamiento de la cefalea tensional.
Hasta la fecha, únicamente se han encontrado cuatro trabajos indexados que estudien el efecto de esta técnica. Todos ellos lo hacen aplicando la fibrolisis en regiones de las extremidades obteniendo resultados beneficiosos sobre el dolor y la movilidad de las articulaciones sobre las que se aplica (40–43).

JUSTIFICACIÓN DEL PROYECTO

La cefalea tensional es una patología muy prevalente en la población general que, a pesar de la amplia variedad de técnicas de tratamiento disponibles, en muchos pacientes presenta una clínica persistente y con tendencia a la cronicidad.
En mi experiencia clínica diaria con pacientes afectados de cefalea tensional, muchos de los de ellos manifiestan que la FD es la técnica que está consiguiendo beneficios permanentes en el curso de su sintomatología. La FD, al producir resultados apreciables desde las primeras sesiones, contribuye no sólo a mejorar la situación clínica del paciente, sino a incrementar los resultados del resto de las técnicas y tratamientos aplicados.
Los buenos resultados clínicos de la FD, en esta y en otras patologías, hacen que tenga un auge creciente entre los fisioterapeutas, tanto en el ámbito nacional como en el internacional. No obstante, la efectividad de la FD no ha sido probada en ensayos clínicos sobre la cefalea tensional.
Con este ensayo clínico se pretende objetivar las mejoras clínicas de estos pacientes y verificar si la FD aporta realmente una ventaja suplementaria al tratamiento habitual de la cefalea tensional.

De confirmarse su eficacia, supondría un avance en el tratamiento de los pacientes afectados de cefalea tensional, una patología muy prevalente y discapacitante que, a pesar de los múltiples tratamientos de disponibles, para los fisioterapeutas sigue suponiendo un reto en la práctica clínica diaria.

HIPÓTESIS Y OBJETIVOS

HIPÓTESIS

La técnica de fibrolisis diacutánea sobre las estructuras miofasciales de la región cráneo-cervical en pacientes con cefalea tensional produce una reducción en la frecuencia e intensidad del dolor y una mejora en la función de la región cervical, así como en la discapacidad y calidad de vida percibida por los pacientes.

OBJETIVOS

OBJETIVO PRINCIPAL

- Identificar los resultados clínicos que produce una intervención mediante fibrolisis diacutánea sobre la musculatura de la columna cervical en pacientes con cefalea tensional.

OBJETIVOS SECUNDARIOS

- Describir los efectos clínicos que produce una intervención mediante fibrolisis diacutánea sobre estructuras miofasciales de la región cráneo-cervical en distintos aspectos del dolor en pacientes con cefalea tensional.
- Describir los efectos clínicos que produce una intervención mediante fibrolisis diacutánea sobre estructuras miofasciales de la región cráneo-cervical en la función articulares y muscular de la región cervical en pacientes con cefalea tensional.
- Describir los efectos clínicos que produce una intervención mediante fibrolisis diacutánea sobre estructuras miofasciales de la región cráneo-cervical en la postura de pacientes con cefalea tensional.
- Describir los efectos clínicos que produce una intervención mediante fibrolisis diacutánea sobre estructuras miofasciales de la región cráneo-cervical en la discapacidad en pacientes con cefalea tensional.
- Describir los efectos clínicos que produce una intervención mediante fibrolisis diacutánea sobre estructuras miofasciales de la región cráneo-cervical en la calidad de vida en pacientes con cefalea tensional.
- Relacionar los efectos clínicos anteriormente citados con las características clínicas de los pacientes con cefalea tensional, con el objetivo de establecer reglas de predicción clínica para la técnica de fibrolisis diacutánea en este subgrupo de pacientes.

METODOLOGÍA

DISEÑO

Para alcanzar los objetivos del estudio se diseñará un ensayo clínico aleatorio controlado, que evalúe los efectos clínicos de la aplicación de fibrolisis diacutánea comparándolo con el tratamiento conservador habitual. Dadas sus características se tratará de un estudio analítico, de carácter longitudinal, prospectivo y experimental.

Aspectos éticos

Este proyecto se presentará al Comité Ético de Investigación Clínica de Aragón (CEICA) siendo su aprobación necesaria para continuar con las siguientes etapas del estudio.

Variables de estudio

Variables independientes

- Tratamiento de intervención mediante fibrolisis diacutánea sobre estructuras miofasciales de la región cráneo-cervical en posición decúbito prono.

- Grupo control, que permanecerá el mismo tiempo que el grupo intervención en decúbito supino con condiciones ambientales similares.

VARIABLE	TIPO	INSTRUMENTO	VALOR
Edad	Cuantitativa discreta	Anamnesis	Años
Sexo	Cualitativa nominal		Hombre / Mujer
IMC	Cuantitativa discreta		Km/m^2
Hábitos tóxicos	Cuantitativa nominal		Alcohol / tabaco / drogas
Actividad laboral	Cualitativa nominal		Activo / Parado / Jubilado
Posición actividad laboral	Cualitativa nominal		Sedestación / Bipedestación / Deambulación / Combinado
Horas actividad laboral	Cuantitativa discreta		Horas
Actividad física	Cualitativa nominal		Sí / No
Horas actividad física	Cuantitativa discreta		Horas
Medicación analgésica	Cualitativa nominal		Si / No
Historia cefalea	Cuantitativa continua		Años
Frecuencia cefalea	Cuantitativa discreta		Días
Duración crisis	Cuantitativa discreta		Minutos
Localización	Cualitativa nominal		Frontal / Occipital / Temporal / Parietal
Cualidad del dolor	Cualitativa nominal		Opresivo / Pulsátil / Continuo
Intensidad del dolor	Cuantitativa discreta		EVA (10cm)

Signos acompañantes	Cualitativa nominal		Vómitos / Fonofobia / Fotofobia / Otros / Ninguno
Necesidad de acostarse	Cualitativa nominal		Si / No
Factores desencadenantes	Cualitativa nominal		Si / No
Factores agravantes	Cualitativa nominal		Si / No

VARIABLES DEPENDIENTES

**VARIABLE**	**INSTRUMENTO**
Intensidad Dolor	Escala Visual Analógica (r = 0.94, P < 0.001) (44)
Frecuencia	Hoja de registro
Duración	Hoja de registro
Localización	Hoja de registro (mapa topográfico)
Umbral de dolor a la presión	Algómetro de presión r=0,92 (ICC) (45)
Sensibilidad muscular	Valoración manual (criterios según Langemark y Olesen) (18)
Movilidad de los tabiques intermusculares	Valoración manual
Longitud muscular	Valoración manual
Fuerza de musculatura flexora profunda	Test de flexión cráneo-cervical r=0.63-0.86 (ICC) (46)
Rango de Movimiento cervical	CROM r=0.87-0.94 (ICC) (46)
Posición de cabeza adelantada	Cámara fotográfica Interexaminador r> 0.85 para los tres planos del espacio (47)
Sección transversal de músculos cervicales	Ecógrafo
Discapacidad	Cuestionario HIT-6
Calidad de vida	Cuestionario COOP-WONCA

Las variables se han elegido en base a una revisión realizada en 2013 por J. Abboud en la que seleccionó las variables musculoesqueléticas más utilizadas en la evaluación realizada a pacientes con cefalea tensional en ensayos clínicos (20). Además se han añadido variables que valoren parámetros musculares que puedan ser relevantes, así como calidad de vida y discapacidad para ver el impacto de esta patología en la vida diaria de estos pacientes.

MUESTRA DEL ESTUDIO

TAMAÑO MUESTRAL

Se ha escogido la variable mejora en la frecuencia de cefalea como referencia para el cálculo del tamaño muestral.

El cálculo se ha realizado con el programa online GRANMO 7.12 con el menú de comparación entre dos medias independientes con un contraste unilateral y los siguientes valores: riesgo alfa 0,05; riesgo beta 0,20; para detectar una diferencia igual o superior a 3 unidades; una desviación estándar común de 5 unidades; una razón entre el número de sujetos entre los grupos 1 y estimando un 15% de pérdidas de

seguimiento. El resultado es que se necesitan 41 sujetos por grupo, como en este estudio se dispondrá de dos grupos, son 82 sujetos en total. El reclutamiento de este número de sujetos se prevé factible.
Los datos estadísticos necesarios para realizar el cálculo se han obtenido del estudio de Castien et al. (48) sobre la comparación de la aplicación de técnicas de terapia manual frente al tratamiento médico habitual en pacientes con cefalea tensional crónica, con una población de consultas externas ambulatorias en el área de Holanda.

RECLUTAMIENTO Y OBTENCIÓN DE LA MUESTRA

La participación en el estudio se ofrecerá a pacientes de distintos servicios de atención primaria del área metropolitana de Zaragoza. Se informará del estudio a aquellos pacientes que estando diagnosticados de cefalea tensional por el médico responsable de cada servicio puedan ser susceptibles de beneficiarse de la participación en este estudio.

CRITERIOS DE INCLUSIÓN Y EXCLUSIÓN

Criterios de inclusión:

- Presentar diagnóstico de cefalea tensional episódica o crónica, realizado por un profesional médico del área de neurología, siguiendo los criterios diagnósticos fundamentales para cefalea tensional establecidos en la Clasificación Internacional de Cefaleas (ICHD-III).
- Tener más de 18 años de edad.
- Capacidad para rellenar los cuestionarios.
- Asistencia a todas las sesiones de intervención y valoración.
- Lectura del documento informativo y firma del consentimiento informado.

Criterios de exclusión:

- Implicación en indemnizaciones o litigios por problemas de salud.
- Recibir tratamiento fisioterápico en la región cráneo-cervical en el mes anterior al estudio o durante el mismo. No obstante, se permite que el sujeto continúe con la medicación paliativa que sigue hasta la realización del estudio por razones éticas y para facilitar la precisión del estudio.
- Sujetos con presencia de banderas rojas para las cefaleas (49), así como sujetos que puedan tener enfermedades severas que puedan estar relacionadas con los resultados clínicos: malignidad o historia de cáncer, infección vertebral, tumores o fracturas vertebrales, inestabilidad lumbar, discrasia sanguínea, traumastismo severo en los 3 meses previos, cirugía de cuello en los 12 meses previos, dolor cervical referido de origen visceral.
- Insuficiente comprensión del castellano.
- Cambio de pautas de medicación habituales en el último mes.
- No completar todas las sesiones propuestas.

ALEATORIZACIÓN

Los sujetos serán aleatoriamente asignados a un al Grupo Intervención mediante FD en la región cráneo-cervical (Grupo A) o a un Grupo Control (Grupo B). El proceso de aleatorización se realizará mediante un muestreo aleatorio sistemático, junto a la realización de una lista de números aleatorios (1 y 2) creados a partir de una aplicación informática de aleatorización de números (1 = Grupo A; 2 = Grupo B).

INTERVENCIÓN A REALIZAR

Una vez seleccionada la muestra realizará una evaluación inicial recogiendo datos sobre la Historia Clínica del paciente utilizando las variables de la anamnesis citadas anteriormente. Además, para identificar más características clínicas de la muestra de pacientes con cefalea tensional, junto con la historia clínica, se realizará una

valoración de: el umbral de dolor a la presión en los músculos más relevantes de la región cervical mediante la valoración con un algómetro de presión, rango de movimiento cervical en los tres planos del espacio mediante el aparato de medición para el rango de movimiento cervical CROM y longitud muscular de músculos cervico-escapulares, la posición de cabeza adelantada en sedestación y en bipedestación mediante fotometría, la resistencia de la musculatura flexora mediante el test de flexión-cráneo cervical (50) y el área de sección transversal de la musculatura cervical profunda mediante ecografía.
Ambos grupos asistirán a 3 sesiones en días alternos durante una semana. Cada una de las sesiones constará de una valoración pre-intervención (intensidad, frecuencia y localización del dolor), de una intervención (FD en la región cráneo-cervical / Control) y de una valoración post-intervención (intensidad, frecuencia y localización del dolor). Además, se realizará una última valoración completa 4 semanas después de la última intervención. Esta valoración se realizará durante todo el estudio en la misma franja horaria y será precedida de algunos ejercicios de calentamiento activo por parte del paciente. El tiempo aproximado de cada sesión será de 60 minutos.
Cada sesión de tratamiento del Grupo Intervención consistirá en una sesión de FD en la región cráneo-cervical, durante 30 minutos, la cual se dirigirá principalmente al abordaje de los bordes, las inserciones y el tejido tendinoso insercional de los siguientes músculos: trapecio, paravertebrales, angular de la escápula, esplenio de la cabeza y del cuello, suboccipitales, esternocleidomastoideo y temporal. El paciente será informado que la técnica debe ser en todo momento indolora y sólo se permite una leve sensación punzante durante la tracción con el gancho (por debajo de 3 sobre 10 en una escala EVA). El Grupo Control recibirá las mismas valoraciones y permanecerá el mismo tiempo de tratamiento en decúbito supino sin recibir ninguna intervención.
Se solicitará a los participantes de ambos grupos que no modifiquen pautas ni de tratamiento ni de ejercicio físico habitual, ni de horas de sueño y que, en la medida que puedan, no varíen de manera muy marcada las actividades de su vida diaria durante el periodo que dure el estudio.

RECOGIDA Y ANÁLISIS DE DATOS

Aquellos pacientes de los servicios médicos previamente mencionados, susceptibles de participar en este estudio según el especialista médico correspondiente y que cumplan los criterios de inclusión y exclusión serán derivados para que un fisioterapeuta le facilite la información (oral y escrita) del estudio. Aquellos pacientes que decidan colaborar deberán firmar el consentimiento informado y se les asignará un número correlativo. Este mismo fisioterapeuta se encargará de realizar la valoración inicial, post intervención y de seguimiento manteniéndose cegado sobre el grupo asignado. Esta valoración incluirá: Anamnesis, reparto de cuestionarios, recogida de datos en la hoja de exploración relativos al dolor, exploración de la función articular y muscular de la región cervical, realización de fotografías para analizar la postura y realización de varias ecografías de la musculatura cervical.
Un segundo fisioterapeuta, que será el único con acceso al listado de aleatorización aplicará la técnica de fibrolisis diacutánea a los pacientes asignado al grupo intervención. También registrará las posibles incidencias o complicaciones que hayan podido darse, tales como hematomas, reacciones cutáneas, etc.
Al tratarse de una técnica manual el fisioterapeuta no puede estar cegado a la condición del paciente, pero con este procedimiento se respeta la ocultación de la asignación.

ANÁLISIS ESTADÍSTICO

El estudio estadístico se llevará a cabo con el programa SPSS versión 22.0 para Mac. El nivel de confianza establecido para el análisis de los resultados será del 95%. La persona que analizará los resultados, estará cegada a las intervenciones asignadas a cada sujeto.

Para alcanzar los objetivos del estudio, se realizará un estudio descriptivo, un estudio comparativo, un estudio correlacional y de regresión de los datos recogidos.
Para el análisis descriptivo de las variables cuantitativas se utilizarán los índices de tendencia central (media y mediana) y los índices de dispersión (desviación típica y los valores mínimo y máximo). Para las variables cualitativas, se realizará un estudio de frecuencias para conocer las frecuencias absolutas y relativas, y los porcentajes válidos y acumulativos. En algunos casos, se extraerá la medida de tendencia central moda.
Previo a la realización del estudio comparativo entre ambos grupos, se realizará un estudio para conocer la normalidad y homocedasticidad de las variables de escala del estudio y así poder utilizar las operaciones estadísticas correspondientes.

Plan de trabajo

Etapas de desarrollo

1. Completar la búsqueda bibliográfica: Enero 2015
2. Análisis de la bibliografía y redacción del proyecto: Abril 2015
3. Envío al Comité de Ética: Junio 2015
4. Reclutamiento de sujetos: Septiembre 2015 - Septiembre 2016
5. Análisis de los datos: Noviembre 2016
6. Redacción de los resultados: Febrero 2017
7. Presentación de la tesis: Primavera de 2017

Lugar de realización del proyecto

El estudio se realizará en las instalaciones de la Facultad de Ciencias de la Salud de la Universidad de Zaragoza.

Listado de referencias

1. Fernández-de-Las-Peñas C, Ge H-Y, Alonso-Blanco C, González-Iglesias J, Arendt-Nielsen L. Referred pain areas of active myofascial trigger points in head, neck, and shoulder muscles, in chronic tension type headache. J Bodyw Mov Ther. 2010 Oct;14(4):391–6.

2. Gemma V E-L, Antonia G-C. Efficacy of manual and manipulative therapy in the perception of pain and cervical motion in patients with tension-type headache: a randomized, controlled clinical trial. J Chiropr Med. 2014 Mar;13(1):4–13.

3. Ashina S, Bendtsen L, Ashina M. Pathophysiology of migraine and tension-type headache. Tech Reg Anesth Pain Manag. 2012;16:14–8.

4. Ferrante T, Manzoni GC, Russo M, Camarda C, Taga A, Veronesi L, et al. Prevalence of tension-type headache in adult general population: the PACE study and review of the literature. Neurol Sci. 2013 May;34 Suppl 1:S137–8.

5. Chowdhury D. Tension type headache. Ann Indian Acad Neurol. 2012 Aug;15(Suppl 1):S83–8.

6. WHO | Headache disorders [Internet]. World Health Organization; [cited 2015 May 19]. Available from: http://www.who.int/mediacentre/factsheets/fs277/en/

7. Steiner TJ, Martelletti P. Aids for management of common headache disorders in primary care. J Headache Pain. 2007 Oct;8 Suppl 1:S2.

8. The International Classification of Headache Disorders, 3rd edition (beta version). Cephalalgia. 2013 Jul;33(9):629–808.

9. Lyngberg AC, Rasmussen BK, Jørgensen T, Jensen R. Has the prevalence of migraine and tension-type headache changed over a 12-year period? A Danish population survey. Eur J Epidemiol. 2005 Jan;20(3):243–9.

10. Vos T, Flaxman AD, Naghavi M, Lozano R, Michaud C, Ezzati M, et al. Years lived with disability (YLDs) for 1160 sequelae of 289 diseases and injuries 1990-2010: A systematic analysis for the Global Burden of Disease Study 2010. Lancet. 2012;380(9859):2163–96.

11. Stovner L, Hagen K, Jensen R, Katsarava Z, Lipton R, Scher A, et al. The global burden of headache: a documentation of headache prevalence and disability worldwide. Cephalalgia. 2007 Mar;27(3):193–210.

12. Lyngberg AC, Rasmussen BK, Jørgensen T, Jensen R. Prognosis of migraine and tension-type headache: a population-based follow-up study. Neurology. 2005 Aug 23;65(4):580–5.

13. Guerrero ÁL, Rojo E, Herrero S, Neri MJ, Bautista L, Peñas ML, et al. Characteristics of the first 1000 headaches in an outpatient headache clinic registry. Headache. 2011 Feb;51(2):226–31.

14. Gracia-Naya M. [The importance of headaches in neurology clinics. Study groups of neurologists of Aragon]. Rev Neurol. 1999;29(5):393–6.

15. Linde M, Gustavsson A, Stovner LJ, Steiner TJ, Barré J, Katsarava Z, et al. The cost of headache disorders in Europe: the Eurolight project. Eur J Neurol. 2012 May;19(5):703–11.

16. Bendtsen L, Jensen R, Jensen NK, Olesen J. Pressure-controlled palpation: a new technique which increases the reliability of manual palpation. Cephalalgia. 1995 Jun;15(3):205–10.

17. Jensen R, Rasmussen BK, Pedersen B, Olesen J. Muscle tenderness and pressure pain thresholds in headache. A population study. Pain. 1993 Feb;52(2):193–9.

18. Langemark M, Olesen J. Pericranial tenderness in tension headache. A blind, controlled study. Cephalalgia. 1987 Dec;7(4):249–55.

19. Ashina M. Neurobiology of chronic tension-type headache. Cephalalgia. 2004 Mar;24(3):161–72.

20. Abboud J, Marchand A-A, Sorra K, Descarreaux M. Musculoskeletal physical outcome measures in individuals with tension-type headache: a scoping review. Cephalalgia. 2013 Dec;33(16):1319–36.

21. Ashina M, Bendtsen L, Jensen R, Sakai F, Olesen J. Muscle hardness in patients with chronic tension-type headache: relation to actual headache state. Pain. 1999 Feb;79(2-3):201–5.

22. Ashina S, Bendtsen L, Lyngberg AC, Lipton RB, Hajiyeva N, Jensen R. Prevalence of neck pain in migraine and tension-type headache: A population study. Cephalalgia. 2014 May 22;

23. Alonso-Blanco C, Fernández-de-las-Peñas C, Fernández-Mayoralas DM, De-la-Llave-Rincón AI, Pareja JA, Svensson P. Prevalence and anatomical localization of muscle referred pain from active trigger points in head and neck musculature in adults and children with chronic tension-type headache. Pain Med. 2011 Oct;12(10):1453–63.

24. Fernández-de-Las-Peñas C, Alonso-Blanco C, Cuadrado ML, Pareja J a. Myofascial trigger points in the suboccipital muscles in episodic tension-type headache. Man Ther. 2006 Aug;11(3):225–30.

25. Ashina S, Babenko L, Jensen R, Ashina M, Magerl W, Bendtsen L. Increased muscular and cutaneous pain sensitivity in cephalic region in patients with chronic tension-type headache. Eur J Neurol. 2005 Jul;12(7):543–9.

26. Katsarava Z, Dzagnidze A, Kukava M, Mirvelashvili E, Djibuti M, Janelidze M, et al. Primary headache disorders in the Republic of Georgia: prevalence and risk factors. Neurology. 2009 Nov 24;73(21):1796–803.

27. Jensen R, Bendtsen L, Olesen J. Muscular factors are of importance in tension-type headache. Headache. 1998 Jan;38(1):10–7.

28. Fernández-de-Las-Peñas C. Physical therapy and exercise in headache. Cephalalgia. 2008 Jul;28 Suppl 1:36–8.

29. Bendtsen L, Fernández-de-la-Peñas C. The role of muscles in tension-type headache. Curr Pain Headache Rep. 2011 Dec;15(6):451–8.

30. Bezov D, Ashina S, Jensen R, Bendtsen L. Pain perception studies in tension-type headache. Headache. 2011 Feb;51(2):262–71.

31. Shah JP, Phillips TM, Danoff J V, Gerber LH. An in vivo microanalytical technique for measuring the local biochemical milieu of human skeletal muscle. J Appl Physiol. 2005 Nov;99(5):1977–84.

32. Loder E, Rizzoli P. Tension-type headache. BMJ. 2008 Jan 12;336(7635):88–92.

33. Kristoffersen ES, Grande RB, Aaseth K, Lundqvist C, Russell MB. Management of primary chronic headache in the general population: the Akershus study of chronic headache. J Headache Pain. 2012 Mar;13(2):113–20.

34. Kaniecki RG. Tension-type headache. Continuum (Minneap Minn). 2012 Aug;18(4):823–34.

35. Bendtsen L, Evers S, Linde M, Mitsikostas DD, Sandrini G, Schoenen J. EFNS guideline on the treatment of tension-type headache - report of an EFNS task force. Eur J Neurol. 2010 Nov;17(11):1318–25.

36. Tricás-Moreno JM, Lucha-López O, Duby P. Fibrolisis diacutánea: según el concepto de Kurt Ekman. 1ª Edición. Asociación Española de Fibrolisis Diacutánea, editor. 2010.

37. Ekman K. Eine neue methode der fibrolyse zur unterstützung der manuellen therapie. Man Medizin. 1972;10:3–6.

38. Burnotte J, Duby P. Fibrolyse Diacutanée et algies de l'appareil locomoteur. Kinésithérapie Sci. 1988;271:16–8.

39. Colombo I, Ekman K. La fibrolisi diacutenea nuovo mezzo diagnostico e terapeutico in fisiatria. Eura Medicophys. 1968;4:29–36.

40. Barra ME, López C, Fernández G, Murillo E, Villar E, Raya L. The immediate effects of diacutaneous fibrolysis on pain and mobility in patients suffering from painful shoulder: a randomized placebo-controlled pilot study. Clin Rehabil. 2011;25(4):339–48.

41. Barra López ME, López de Celis C, Fernández Jentsch G, Raya de Cárdenas L, Lucha López MO, Tricás Moreno JM. Effectiveness of Diacutaneous Fibrolysis for the treatment of subacromial impingement syndrome: a randomised controlled trial. Man Ther. 2013 Oct;18(5):418–24.

42. Loro C, Lucha O, Caudevilla S, Marín E, Tricás JM, Estébanez E. Fibrolisis Diacutánea: tratamiento de un caso. Cuest Fisioter. 2000;14:9–15.

43. Tricás JM, Lucha O, García Bernabé. Tratamiento de un hombro congelado. Fundamentación teórica y caso clínico. Ter Man Venez. 1998;1:22–30.

44. Hawker GA, Mian S, Kendzerska T, French M. Measures of adult pain: Visual Analog Scale for Pain (VAS Pain), Numeric Rating Scale for Pain (NRS Pain), McGill Pain Questionnaire (MPQ), Short-Form McGill Pain Questionnaire (SF-

MPQ), Chronic Pain Grade Scale (CPGS), Short Form-36 Bodily Pain Scale (SF. Arthritis Care Res (Hoboken). 2011 Nov;63 Suppl 1:S240–52.

45. Fischer AA. Pressure algometry over normal muscles. Standard values, validity and reproducibility of pressure threshold. Pain. 1987 Jul;30(1):115–26.

46. Jørgensen R, Ris I, Falla D, Juul-Kristensen B. Reliability, construct and discriminative validity of clinical testing in subjects with and without chronic neck pain. BMC Musculoskelet Disord. 2014 Dec 4;15(1):408.

47. Ruivo RM, Pezarat-Correia P, Carita AI. Intrarater and interrater reliability of photographic measurement of upper-body standing posture of adolescents. J Manipulative Physiol Ther. 2015 Jan;38(1):74–80.

48. Castien RF, van der Windt DAWM, Dekker J, Mutsaers B, Grooten A. Effectiveness of manual therapy compared to usual care by the general practitioner for chronic tension-type headache: design of a randomised clinical trial. BMC Musculoskelet Disord. 2009 Jan;10:21.

49. Fernández-de-las-Peñas C, Arendt-Nielsen L, Gerwin RD. Fisiopatología, diagnóstico y tratamiento. Cefalea Tensional y de Origen Cervical. Barcelona: Elsevier; 2010.

50. Jull G, Barrett C, Magee R, Ho P. Further clinical clarification of the muscle dysfunction in cervical headache. Cephalalgia. 1999 Apr;19(3):179–85.
